# Locating the microbes along the maize root system under nitrogen limitation: a root phenotypic approach

**DOI:** 10.1093/aob/mcaf185

**Published:** 2025-08-12

**Authors:** Tania Galindo-Castañeda, Elena Kost, Elena Giuliano, Rafaela Feola Conz, Johan Six, Martin Hartmann

**Affiliations:** Institute of Agricultural Sciences, Department of Environmental Systems Science, ETH Zurich, 8092 Zurich, Switzerland; Institute of Agricultural Sciences, Department of Environmental Systems Science, ETH Zurich, 8092 Zurich, Switzerland; Institute of Agricultural Sciences, Department of Environmental Systems Science, ETH Zurich, 8092 Zurich, Switzerland; Institute of Agricultural Sciences, Department of Environmental Systems Science, ETH Zurich, 8092 Zurich, Switzerland; Institute of Agricultural Sciences, Department of Environmental Systems Science, ETH Zurich, 8092 Zurich, Switzerland; Institute of Agricultural Sciences, Department of Environmental Systems Science, ETH Zurich, 8092 Zurich, Switzerland

**Keywords:** *Zea mays*, root architecture, root anatomy, root phenotyping, root microbiome, nitrogen limitation, mesocosms, prokaryotes, greenhouse experiment

## Abstract

**Background:**

A major challenge in agriculture is the low nitrogen (N) uptake efficiency of crops, which poses environmental and economic costs. Root adaptive architectural and anatomical phenotypes in synergy with root microbes could be a promising approach to improve plant N uptake. However, little is known about such synergies. Here, we aimed to characterize the spatial distribution of the root prokaryotes of maize (*Zea mays*) under low N in 30-L mesocosms, where root architecture and anatomy are freely expressed, searching for correlations between prokaryotic genus abundance and ten phenotypes.

**Methods:**

We studied the root prokaryotic community of 4-week-old plants growing in 30-L mesocosms under low N using two sandy soil mixtures. We collected root, rhizosphere and bulk soil samples at various locations, including depths (0–20, 20–70, 70–150 cm), root classes (lateral and axial) and root types (seminal and crown). We measured plant growth response to low N availability and performed 16S rRNA gene metabarcoding on extracted DNA.

**Key Results:**

Sampling location was the third most important factor after soil mixture and compartment, explaining ∼5 % of the variance in root prokaryotic diversity. Seminal roots (0–20 cm depth), shallow crown roots (0–20 cm) and deep crown roots (20–150 cm) showed well-separated root microbial communities. Lateral root branching density (LRBD) explained 10 % of this variance in the rhizosphere and the root tissue. We identified prokaryotic genera specific to depth, soil–root compartment, root class and type under LN. Moreover, architectural phenotypes LRBD and lateral root length significantly correlated with the abundance of 37 genera.

**Conclusions:**

We highlight the importance of sampling location and architectural traits that may be associated with the microbial cycling of soil N. The exploration of synergies between root traits and microbes that participate in the N cycle has the potential to increase sustainability in agriculture.

## INTRODUCTION

Nitrogen (N) uptake efficiency in cereals usually does not exceed 50 % of applied N (Raun and Johnson, 1999). This leads to economic losses and environmental pollution in industrialized and intensive agricultural systems due to excess application of fertilizers, and to food insecurity in smallholder farming and extensive agricultural systems due to lack of access to fertilizers (Robertson and Vitousek, 2009). Two approaches proposed to tackle this problem are (1) breeding for root architectural and anatomical phenotypes linked to increased N uptake (Wang *et al*., 2005; Liu *et al*., 2008; Gaudin *et al*., 2011; Lynch, 2013) and (2) the promotion of microbes that participate in soil N cycling by making N more readily available to plants in the rhizosphere and root endosphere (Jackson *et al*., 2012; Coskun *et al*., 2017). We propose that these two approaches can be used in combination to improve N uptake efficiency in crops (Galindo-Castañeda *et al*., 2022).

The maize root system is composed of embryonic and postembryonic roots. A primary root emerges from the seed, anchoring the plant at germination. Several lateral roots emerge from this primary root, and others, the seminal roots, emerge from the embryo, providing nutrients in the first days of plant growth (Hochholdinger, 2009). A few days after germination, crown roots start emerging in whorls from the underground portion of the shoot tissue. They then build the bulk of the root scaffolds from which lateral roots are produced to sustain plant growth and nutrient uptake (Hochholdinger, 2009). Root architecture (the 3-D arrangement of root organs in space and time) and anatomy (the internal distribution of tissues inside the root organs) exhibit notable natural variation in maize (Trachsel *et al*., 2011; Burton *et al*., 2015). This variation provides adaptability under contrasting environmental conditions (Lynch, 2019). Root architectural phenotypes adaptive for low N (LN) in maize include a high number of seminal roots, fewer but longer lateral roots from both seminal and crown roots, steep rooting angle and fewer crown roots. Root anatomical phenotypes such as long, dense root hairs, greater cortical aerenchyma formation, greater cortical senescence, reduced number of cell files and larger cortical cells have been associated with improved tolerance to LN in maize (Lynch *et al*., 2023).

Little is known about how these adaptive root phenotypes interact with microbes participating in the N cycle under N limitation. Recent research by He *et al*. (2024) showed that bacteria from the genus *Massilia* are associated with increased tolerance to LN levels. This association occurs via lateral roots of maize by increasing lateral root branching density (LRBD). Furthermore, the microbial partnership between maize and *Azospirillum brasilense* can account for up to 25 % savings in N fertilization (Hungria *et al*., 2022). Despite these advances, many gaps remain in this field, as we have highlighted in recent perspectives (Galindo-Castañeda *et al*., 2022, 2024). In our research we aim to understand how interactions between adaptive root traits for LN interact with microbes.

One of the first steps in understanding the interactions between adaptive maize architectural and anatomical phenotypes and microbes is to discern the spatial distribution of microbes along the root system under abiotic stress. However, studying the root phenome and the root microbiome on the same individual plants is difficult. Typically, greenhouse plants are grown in small pots that limit the space for root architectural and anatomical expression (Rüger *et al*., 2021), or in large containers that limit the sampling of rhizosphere for microbiome studies due to logistic limitation in handling large volumes of soil and excavating roots. Despite these challenges, the role of root hairs and lateral root branching has been investigated under limiting N conditions in maize. For example, it is known that the maize root microbiome varies in maize mutants with reduced lateral roots or root hairs (Wang *et al*., 2024), or that gradients in the abundance of taxa exist along the root tips (Rüger *et al*., 2021). Also, greater heritability was found in the rhizosphere microbial communities compared with the endosphere’s, and this heritability was increased under LN in maize (He *et al*., 2024). Furthermore, the genera *Bacillus*, *Chthoniobacter*, *Flavisolibacter*, *Lysobacter*, *Microvirga*, *Sphingobium* and *Sphingomonas* have been found abundant in maize rhizosphere growing under LN (He *et al*., 2024). In addition, a gradient in root microbiome composition has been reported between the root tips and ∼7 cm towards the base of the roots under optimal growth conditions (Rüger *et al*., 2021). Some key taxa have been identified in different root classes and compartments, such as lateral roots, cortex and stele (Wang *et al*., 2024). However, the scales of such studies are still millimetric and focused on root tips growing in small soil containers. A broader understanding of microbiome distributions along entire root systems, covering larger soil volumes and incorporating soil vertical and horizontal gradients, would significantly advance our understanding of interactions between root phenotypes and microbes.

Microbial functions in the N cycle might be affected by N availability and by adaptive root traits (Galindo-Castañeda *et al*., 2022, 2024; see a detailed overview in Tables 2 and 3 of Lynch et al., 2023). For example, high LRBD produces increased carbon exudation and microbial attachment points, as well as steeper gradients of N concentration in the horizontal plane. This could lead to a greater abundance of N-cycling microbial guilds when enough N is available in the soil matrix. Conversely, greater LRBD could lead to a reduction of such guilds when N is critically depleted due to plant–microbe competition for N. Changes in N-cycling microbial guilds, resulting from contrasting N inputs and root phenotypes, may be reflected in changes in microbial diversity under N limitation at different locations along the root systems.

Here, we describe the response of a maize genotype and its associated root microbiome to contrasting N availabilities using 1.5-m tall, 30-L maize mesocosms. These mesocosms have been previously utilized in maize root phenomics (e.g. Jaramillo *et al*., 2013; Schneider *et al*., 2021) and for fungal colonization studies (Galindo-Castañeda *et al*., 2019). The mesocosms allow the testing of simple hypotheses regarding the role of root phenotypes in plant adaptation to stress by simulating the same soil volume a maize plant occupies in the field 4 weeks after planting (Galindo-Castañeda *et al*., 2018). This enables a comprehensive expression of root architecture and anatomy. We provided either high (H) or low (L) N fertilization as fertigation to plants growing in two separate soil mixtures, both of which could potentially be used for future experiments exploring associations between root phenotypes and root microbiomes.

We expected to see a clear differentiation of microbial communities from soil to plant, with a decrease in root prokaryotic alpha diversity and a significant change in beta diversity across the bulk soil, rhizosphere and root microhabitats. We hypothesized that prokaryotic communities in root tissue, rhizosphere and bulk soil would change along different sampling locations in a soil mixture- and N-dependent way. Based on this, we aimed to determine a representative sampling location of the entire root system for future experiments using a similar system. Furthermore, we intended to identify indicator bacterial genera for specific compartments or N treatments across the two soil mixtures. We also investigated the ecological processes driving the assembly of microbial communities along the root system by comparing phylogenetic beta diversity across five different locations. Finally, among the four architectural and six anatomical root phenotypes measured, we anticipated finding significant correlations with genus abundance under the different N availability levels.

## MATERIALS AND METHODS

### Experimental system

**Fig. 1.**
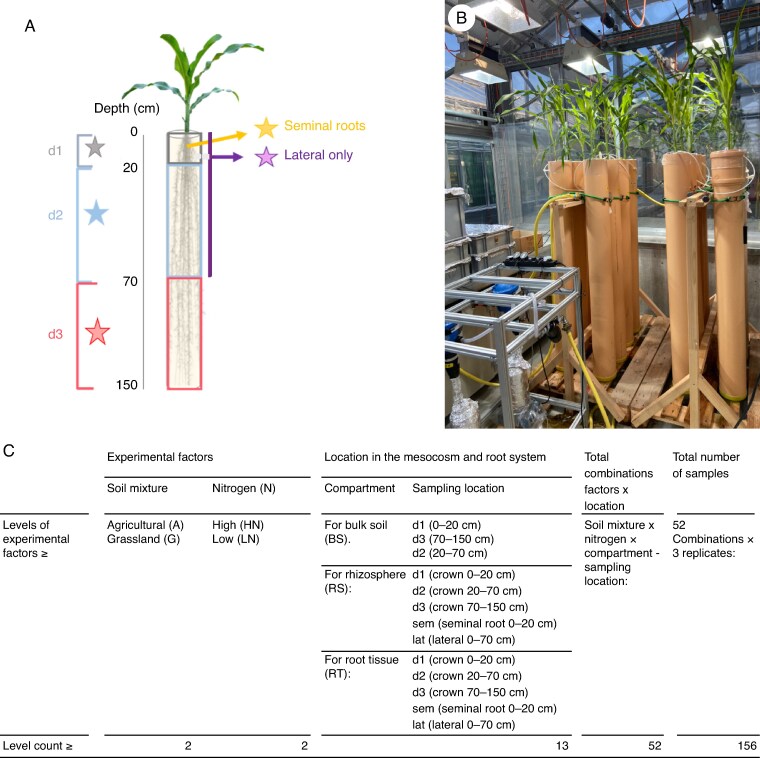
Mesocosms to study root microbiome in maize plants. (A) Overview of the root sampling locations for microbiome profiling. Roots collected at each depth (d1, d2, d3) were crown only and included axial and lateral roots. Stars highlight the differences in sampling location for biodiversity analyses. (B) Image of the mesocosm with irrigation system and nutrient solution source in the greenhouse. More details on this system and sampling can be found as a Supplementary Data Video. (C) Experimental factors, sampling locations and their combinations that resulted in the total sample size for biodiversity analyses of the present study. Note that data coming from one sample (corresponding to root tissue of the grassland soil at d3) had to be discarded due to low read count (<10 % of the mean read count).

Mesocosm systems were established in a greenhouse at the research facilities of ETH in Lindau, Switzerland (47°26′58.6″N, 8°40′57.2″E), similar to the previously described system (Jaramillo *et al*., 2013), between June and August of 2020. Briefly, 1.5-m tall PVC pipes with an internal diameter of 14.5 cm were lined with a polyethylene plastic tubular bag (500 mm diameter and 0.1 mm thick; Innopack, Villmergen, Switzerland), with drainage allowed with two 2-cm parallel linear cuts in the bottom of the bag and filled with two types of mixture, both containing 4-mm-sieved soil (Fig. 1A, B). Grassland soil mixture (referred to in the figures as G) contained 5 % of a silt–loam soil coming from an N-depleted natural grassland provided in March 2020 by the Swiss Federal research centre for agriculture Agroscope (Zurich, Switzerland), which had been collected in their premises (47°25′44.62″N, 8°30′59.73″E). This soil had a total mineral N content of 66.728 µg g^−1^ dry soil. Agricultural soil mixture (referred to as A in the figures), contained 15 % of an agricultural loam soil provided by the horticultural company Ricoter (Frauenfeld, Switzerland). The soil was collected from different sugar beet fields distributed across all eastern Switzerland from October 2019 to January 2020. The soil coming from all these fields was processed in a factory in Frauenfeld (47°33′14.93″N, 8°52′5.74″E) in February 2020 and sold to us. This soil had mineral N content of 102.37 µg g^−1^ dry soil (analyses results are in Supplementary Data Table S1). Each soil was mixed with 50 % v/v 0.7–1.2 mm quartz sand (Carlo Bernasconi AG, Zurich, Switzerland), 10 % 2–6 mm perlite (Ricoter, Frauenfeld, Switzerland) and 35 % (mixture G) or 25 % (mixture A) 0.4–1.2 mm vermiculite (gvz-rossat AG, Otelfingen, Switzerland). The purpose of preparing a sandy mixture was to facilitate root washing and phenotyping. The percentage ranges 5–20 % of soil in similar mixtures have been previously used in root maize phenotyping research (Jaramillo *et al*., 2013; Schneider *et al*., 2021). We used different percentages of soil for the G and the A mixture to match the availability of such soil for future experiments. Note that access to and excavation of soil in Switzerland is highly regulated. Therefore, with this experiment we aimed to provide soil percentages that could later be used in larger experiments (e.g. more genotypes, more replicates), to follow up on the results presented here. To ensure a similar bulk density across mesocosms, the volume of the media in the cylinders was controlled using 5-L filling flasks to measure the exact number of litres filled into each column. HN and LN supplies were provided to the columns using a modified Barber’s nutrient solution for hydroponic maize growth (York *et al*., 2016; Yang, 2017) containing the following elemental composition (in µm): 6100 (HN) and 155 (LN) NO_3_-N, 100 (HN) and 5 (LN) NH_4_-N, 3000 (HN) and 1500 (LN) K; 3250 (HN) and 825 (LN) Ca; 500 P; 2000 Mg; 3500 (HN) and 3000 (LN) S; 18.3 (HN) and 518 (LN) Cl; 46.2 B; 9.1 Mn; 0.8 Zn; 0.3 Cu; 0.1 Mo; 50 Fe. Four days after planting, 250 mL of the nutrient solution was provided per column and from day 12 onwards each column received 250 mL of nutrient solution (pH 5.5) twice a day using a Dosatron (Dosatron International, Tresses, France) connected to a drip irrigation system, maintaining the columns at 60–90 % of field capacity.

### Plant material, greenhouse conditions and experimental design

The recombinant inbred maize line IBM181 (from intermated B73 × Mo17; Senior *et al*., 1996) was selected due to its intermediate crown root number, root biomass, axial root length and lateral root length as previously reported for similar growth conditions (Yang, 2017). Seeds were treated with 50 % bleach solution for 5 min then rinsed three times with sterile distilled water (sdH_2_O) and left to imbibe for 4 h in sdH_2_O. The cup with the soaked seeds was put into a 60 °C water bath for 5 min and again rinsed three times with sdH_2_O. Autoclaved germination paper (Anchor Paper Company, Saint Paul, MN, USA) was saturated with 0.5 mm CaSO_4_ in a tray, and the seeds were placed 3 cm apart from each other. The paper was rolled and placed upright in 1-L beakers halfway filled with the CaSO_4_ solution and incubated at 27 °C in the dark until emergence. Twenty-four hours before planting, the columns were saturated to field capacity with 4 L of the prepared nutrient solution. Three seedlings per mesocosm were planted and reduced to one plant after 4 d. Columns were arranged in a split randomized design, split by N levels and randomized for soil mixtures using three replicates per treatment combination for a total of 12 mesocosms (Fig. 1C). The greenhouse was set to 25 °C, 60 % humidity and 14/10 h photoperiod with supplementary illumination (400-W metal halide lamps; Hugentobler Spezialleuchten AG, Weinfelden, Switzerland) to obtain a final light input of 400–1050 µmol photons (m^2^ s^−1^) at noon. Harvest occurred 31–32 d after sowing.

### Sample collection and measurements at harvest

The entire shoot was harvested at the end of the 4 weeks, just before transferring the mesocosm column to the root washing room, where the plastic bag filled with soil was pulled out of the PVC pipe and opened with clippers to sample the root system. Around three or four root segments of ∼10 cm with their adhering soil were sampled at five different points within the root system as shown in Fig. 1 (crown 0–20 cm, crown 20–70 cm, crown 70–150 cm, seminal 0–20 cm and lateral 0–70 cm) and directly cooled for microbiome profiling. Our selection of sampling locations was based on two primary criteria: their representativeness of the entire root system and the practical feasibility of sampling them during a typical harvest day in this large experimental system. Around 50 g of bulk soil of the mesocosm system without visible influence of roots was collected at three different depths (0–20, 20–70 and 70–150 cm) and put into zip-lock bags (Fig. 1A). The remaining root system from the mesocosm was gently washed with a hose using a support to trap the root system. Loose roots were collected in a 4-mm sieve. A root fragment 8–12 cm from the stem was clipped in the third crown whorl and preserved in 50 % ethanol for anatomical phenotyping, as previously described for anatomical phenotypes of maize mesocosms (Burton *et al*., 2015). The remaining roots were fractioned into four different depths (0–20, 20–40, 20–70 and 70–150 cm; note that the second depth used for biodiversity had to be split in two due to the large sample size at harvest) and stored in 50 % ethanol solution at 4 °C for root architecture phenotyping. Bulk soil samples and plant shoot tissue were collected to measure N content. A list of the different measurements performed is presented in Table 1.

**Table 1.** Root phenotypes measured, acronyms used in this document, and units.

Plant measurement	Abbreviation	Unit
*Plant growth*		
Shoot dry biomass		g
SPAD		Dimensionless
Total N content		g kg^−1^
Chlorophyll content		µg mL^−1^
*Root architecture*		
Lateral root branching density, count of lateral roots per cm of axial root.	LRBD	Count cm^−1^
Lateral root length. Sum of lengths of all lateral roots. Roots with diameter ≤0.4 mm	LRL	cm
Total root length. Sum of lengths of all primary, secondary, and lateral roots.	TRL	cm
Specific root length. Total root length divided by dry weight of sample biomass	SRL	cm g^−1^
Depth above which 95 % of the root length is located	D_95_	cm
*Root anatomy*		
Root cross-section area	RXSA	mm^2^
Living cortical cell area	LCA	mm^2^
LCA as percentage of total RXSA	pLCA	% of total cross-section area
Total cortical area	TCA	mm^2^
Total stele area	TSA	mm^2^
Aerenchyma area	AA	mm^2^
Root cortical aerenchyma	RCA	% of total cortical area

### Measuring N content in soil and plant shoots

Bulk soil and shoot samples were dried (40 °C) and milled (MM200 mill, Retsch GmbH, Haan, Germany, and ZM200, Retsch GmbH, Haan, Germany for soils and shoots, respectively). Approximately 200 mg of soil samples and 100 mg of shoot samples were weighed into tinfoil cups (Säntis Analytical AG, Teufen, Switzerland). Samples were analysed by combustion with the CN 628 elemental analyser (LECO Corporation, St Joseph, MI, USA). Total shoot N per plant was calculated by multiplying N content by shoot dry weight. Mineral N was measured in soil samples (Mulvaney, 1996) using 10 g of moist bulk soil extracted for 1 h on a horizontal shaker in 50 mL of 2 m KCl and filtered through Whatman^®^ Grade 42 filter papers (GE Healthcare Life Science, Whatman™, Westborough, MA, USA). The filtrate was stored overnight at 4 °C. We measured NH_4_^+^ using reagent A (in g L^−1^: 0.5 sodium nitroprusside, 130 sodium salicylate, 100 sodium citrate and 100 sodium tartrate) and reagent B (60 g L^−1^ sodium hydroxide, 20 mL L^−1^ sodium hypochlorite in 100 mL deionized water). The absorbance at 650 nm (Visible Spectrophotometer V-1200, VWR, Radnor, PA, USA) was read after 1 h. For NO_3_^−^, a pre-made solution (0.5 g vanadium (III) chloride, 0.2 g sulphanilamide and 0.01 g *N*-ethylenediamine dihydrochloride in 200 mL 0.5 m HCl) was added to the samples and stored overnight at room temperature, and the absorbance was measured at 540 nm (Visible Spectrophotometer V-1200, VWR, Radnor, PA, USA). The received values of NH_4_^+^ and NO_3_^−^ concentrations were added to get the total mineral N concentration.

### Chlorophyll content

An extract was prepared following the method from Lisec *et al*. (2006) using 15 g of lyophilized and homogenized shoot sample. Chlorophyll *a* and *b* content in the leaves was measured and calculated as described in Warren (2008) using 200 µL of the extract.

### Root phenotyping

For root architecture analysis, washed roots were imaged using an Epson Expression 11000XL flat-bed scanner (Nagano, Japan) in transparent trays containing a 1-cm layer of water at a resolution of 400 dots per inch. Roots collected at each depth for each treatment were separately scanned and analysed with the WinRhizo v.2020a program (Regent Instruments, Quebec City, Canada). LRBD was measured by manually counting the number of lateral roots on two representative 10-cm segments per sample using ObjectJ (available at https://sils.fnwi.uva.nl/bcb/objectj/) implemented in ImageJ (Schneider *et al*., 2012). The two representative root segments were selected by visually inspecting the entire sample and choosing two segments that exhibited a branching pattern similar to most of the roots in the maize crown; this method has been previously described for root phenotyping studies (e.g. Zhan and Lynch, 2015).

Specific root length (SRL) was obtained by dividing the total root length by the dry weight of the root biomass. Roots were washed to remove ethanol and dried overnight in an oven at 60 °C. D95, defined as the depth above which 95 % of the root length is located, was calculated using linear interpolation of the cumulative total root length per depth (Schenk and Jackson, 2002).

For root anatomy analysis, ethanol-preserved root samples were hand-cross-sectioned with a sharp razor blade. Sections were then imaged under an Olympus AX70 microscope (Olympus, Hachioji-shi, Japan) at ×4 magnification with an Olympus XC10 camera (Olympus). The three best images from each plant were analysed with RootScan v.2.0 (Burton *et al*., 2012) to record root anatomical characteristics. A comprehensive list of the phenotypes measured is provided in Table 1.

### Sample preparation and DNA extraction for microbiome profiling

At harvest, root segments were manually shaken to remove loosely adhering soil, and soil that remained attached was defined as rhizosphere soil. This soil was put in 35 mL of a wash buffer solution (6.75 g of KH_2_PO_4_ and 8.75 g of K_2_HPO_4_ in 1000 mL deionized water and 200 µL Tween 20 added after autoclaving). The sampled roots were vortexed (2 min) and roots were separated from the rhizosphere soil in another tube using sterile tweezers. The rhizosphere soil was centrifuged for 10 min at 4 °C and 4000 *g*; the supernatant was decanted and the pellet stored at −20 °C for DNA extraction. The separated root segments were washed with sterile water, cut into ∼5-mm pieces and stored in a tube at −80 °C for DNA analysis (Lundberg *et al*., 2012; Simmons *et al*., 2018). Bulk soil samples collected in the bags were manually homogenized for 30 s and stored at −20 °C until processing. At processing, 3 g of bulk soil was diluted in 35 mL of the wash buffer solution and vortexed for 2 min. Samples were centrifuged at 4 °C at 4000 *g* for 10 min and stored at −20 °C for DNA extraction. DNA was extracted from bulk soil, rhizosphere soil and root tissue using the DNeasy^®^ PowerSoil^®^ Pro kit (Qiagen, Hilden, Germany) according to the manufacturer's protocol. However, for the soil samples, a bead beating grinder and lysis system (FastPrep-24™ 5G, MP Biomedical, Illkirch Cedex, France) was used for two cycles of 40 s with a 40-s pause in between, instead of the vortex adapter in the second step of the manufacturer’s protocol.

Root DNA was extracted from powdered root tissue, obtained by grinding frozen samples with a Geno/Grinder^®^ 2010 (Horiba, Japan). Instead of centrifuging in steps 8–13 in the manufacturer’s protocol, a vacuum (QIAvac 24 Plus, Qiagen, and Vacuubrand Fluid Aspiration System, BVC Control, Wertheim, Germany) was used for washing DNA. The extracted DNA samples were stored at −18 °C. DNA concentration was measured with a QIAxpert spectrophotometer (Qiagen). DNA samples were normalized, before PCR, to a final concentration of 2 ng µL^−1^ using the QIAgility liquid handling system (Qiagen). The V3–V4 region of the 16S rRNA gene was amplified using the primers 341F (5′-CCTAYGGGDBGCWSCAG-3′) and 806R (5′-GGACTACNVGGGTHTCTAAT-3′) (as published by Frey *et al*., 2016). PCR of the bulk and rhizosphere soil samples was performed in a total reaction mixture volume of 25 µL (5.5 µL ddH_2_O, 1× GoTaq^®^ Colorless Master Mix (Promega, Madison, WI, USA), with 0.8 µL of each primer (341F and 806R, each at 0.4 µM) and 10 ng template DNA). The reaction mixture was prepared using the QIAgility instrument (Qiagen). The PCR consisted of 5 min at 95 °C of initial denaturation, followed by 35 cycles of 40 s at 95 °C for denaturation, 40 s at 58 °C for annealing and 1 min at 72 °C for elongation, followed by 10 min at 72 °C for final elongation (C100 Touch Thermal Cycler, Bio-Rad Laboratories, Hercules, CA, USA). For the root samples, 0.76 µm of mitochondrial peptide nucleic acid (mPNA) (5′-GGCAAGTGTTCTTCGGA-3′) and 0.76 µm of plastid PNA (pPNA) (5′-GGCTCAACCCTGGACAG-3′) (Pna Bio, Thousand Oaks, CA, USA) clamps were additionally added to the reaction mixture to a total reaction mixture volume of 25 µL (Lundberg *et al*., 2012; Johnston-Monje *et al*., 2016). PNA clamps were used to block the amplification of the 16S rRNA gene of chloroplasts and mitochondria as described in Lundberg *et al*. (2013). The PCR for the root tissue DNA was modified in reference to the soil PCR to 40 cycles of denaturation, and 58 °C for annealing. PCR performance was checked using gel electrophoreses gel with QIAxcel Advanced (Qiagen) for each PCR replicate. Amplicons were pooled and sent to the Functional Genomics Center Zurich (FGCZ, Zurich, Switzerland) for library generation using the Truseq DNA Nano kit (Illumina Inc., San Diego, CA, USA) and sequencing on the Illumina MiSeq platform with the v3 chemistry (paired-end 300 bp).

### Bioinformatics

Amplicon sequencing data were processed primarily using VSEARCH v.2.30.0 (Rognes *et al*., 2016). Initially, PhiX sequences were removed with Bowtie2 v.2.5.4 (Langmead and Salzberg, 2012), and PCR primers were trimmed using Cutadapt v.5.1 (Martin, 2011), allowing for one mismatch. Paired-end reads were then merged using VSEARCH's *fastq-mergepairs* function. Low-quality sequences were filtered with VSEARCH's *fastq_filter* function, allowing a maximum of one expected error. Dereplication was performed using VSEARCH's *derep_fulllength* function, and singletons were removed. Dereplicated reads were delineated into amplicon sequence variants (ASVs) by running the UNOISE algorithm (VSEARCH's *cluster_unoise* function) with an alpha of 2 and a minimum size of 8 (Edgar, 2016; Callahan *et al*., 2017). Potential chimeric reads were detected and removed using the UCHIME algorithm (Edgar *et al*., 2011) via VSEARCH's *uchime3_denovo* function. The remaining sequences were checked for ribosomal signatures with Metaxa2 v.2.2.3 (Bengtsson-Palme *et al*., 2015), and unconfirmed sequences were removed. Quality-filtered sequences from each sample were then aligned against verified sequences using VSEARCH's *usearch_global* function (maxaccepts = 20, maxrejects = 20). Finally, ASV sequences were taxonomically classified using the SINTAX algorithm implemented in VSEARCH (Edgar, 2016) against the SILVA v.138.2 database (Pruesse *et al*., 2007) with a confidence cutoff of 80 %.

### Statistical analysis

#### Plant and soil measurements

For normally distributed data *t*-tests were performed, and for non-normally distributed data Wilcoxon tests were used to quantify the effect of N treatment on D95, shoot dry weight, SPAD (Soil–Plant Analysis Development meter, Konica Minolta, Tokyo, Japan), total N per kilogram of plant, total N per plant, chlorophyll content, root anatomical phenotypes (RXSA, LCA, pLCA, AA, RCA, TCA, TSA; see Table 1 for definitions of abbreviations), and crown root number. These analyses were conducted separately for each soil mixture (A and G). The effects of N treatment and depth on architectural phenotypes (TRL, LRL, LRBD, SRL), root dry weight, total N and mineral N were analysed with a two-way ANOVA followed by Tukey HSD post hoc tests, performed separately for each soil mixture. Therefore, the effects of depth and N were considered as overall comparisons for each soil mixture. Data were examined for normal distribution using QQ plots and the Shapiro–Wilk test, and for homogeneity of variance using Levene's test. Non-normally distributed data were arcsine-transformed using the bestNormalize package v.1.9.1 (Peterson and Cavanaugh, 2020) and subsequently analysed with a two-way ANOVA. All statistical analyses were conducted with R version 4.5 (R Core Team, 2019) in RStudio. The *dplyr* package v.1.1.4 (Wickham *et al*., 2025) and *tidyr* package v.1.3.1 (Wickham *et al*., 2019) were utilized for data preparation and organization at several steps during the analyses. In all our analyses, *P*-values <0.05 were considered statistically significant.

#### Prokaryotic biodiversity

To inspect the sequencing depth, we calculated rarefaction curves using the *rarecurve* function from the vegan package v.2.7-1 (Supplementary Data Fig. S1; Oksanen *et al*., 2013; Schloss, 2024). Rarefaction was performed 100 times to the read number of the sample with the lowest number of reads (44 432). Both alpha and beta diversity metrics were then calculated on the medians of the 100 subsampled matrices. Alpha diversity was assessed by calculating observed richness (e.g. ASV count), Shannon diversity index and inverse Shannon index of ASVs based on the median abundances of the subsampled ASV matrices, using the *rarefy*, *specnumber* and *diversity* functions from vegan. Beta diversity was assessed with Bray–Curtis dissimilarities. First, differences in beta diversity across the experimental factors were visually examined using principal coordinate analysis (PCoA) (Gower, 2015) to elucidate the major variance components, employing the *cmdscale* function in vegan. Then, PERMANOVA (Anderson, 2001) and PERMDISP (Anderson, 2006) were used to quantify the effects of soil mixture, N, compartment, and sampling location with the *adonis2* function from vegan. Furthermore, we performed a canonical analysis of principal coordinates (CAP) (Anderson and Willis, 2003) to constrain beta diversity by significant factors, such as N and sampling location, using the *CAPdiscrim* function in the *BiodiversityR* package v.2.17-2 (Kindt and Coe, 2005). We further split the database by soil mixture and compartment to identify N- and sampling point-sensitive ASVs and genera.

#### Taxon-level response to N and sampling location

To identify ASVs associated with each combination of soil mixture, N and compartment, we followed two approaches using the matrix containing the median read counts of ASVs from the 100-fold subsampled matrices (see above for details). First, indicator species analyses were performed on the entire, filtered dataset. This was implemented with the *multipatt* function in the *indispecies* package v.1.8.0 (Dufrêne and Legendre, 1997). The matrix for this analysis was pre-filtered by removing (1) rare ASVs with a relative abundance ≤0.01 %; (2) sparse ASVs not occurring in at least three samples; and (3) outlier ASVs that exceeded the abundance in the second most abundant sample by 100-fold. Second, ASV-level PERMANOVAs were applied on datasets split by soil mixture and compartment (rhizosphere (RS) and root tissue (RT)). For these PERMANOVAs, filters were applied to the split datasets (soil mixture × compartment subsets); therefore, the relative abundance filter was based on ASVs present within each specific soil mixture × compartment subset. We calculated *P* and *F* values per ASV using the *adonis2* function with 999 permutations, calculating Euclidean distances and outputting the results by terms to identify ASVs responsive to the different factors entered in the model (median abundance ∼ soil_mixture × N × compartment × sampling_location). The resulting *P*-, *F*- and *q*-values (see below) were then compiled into a summary dataset (Supplementary Data File S1). Next, the subsampled matrix was aggregated by the total sum of the read counts by genus to perform genus-level PERMANOVAs. These analyses allowed us to cover a larger proportion of the biodiversity (∼15.0 % of total ASVs) compared with ASV-level PERMANOVAs (∼1.2 % of total ASVs). Both PERMANOVAs and indicator species analyses were corrected for multiple hypothesis testing using the false discovery rate (FDR) method as described by Storey (2002), implemented in the q value package v.2.40.0 (Storey *et al*., 2025). For visualizing the indicator species results, we used the *euler* function from the eulerr package v.7.0.2 (Larsson, 2024) and the *venn* function from the *venn* package v.1.12 (Dusa, 2024). Bar charts were used for PERMANOVA results. Additionally, to simplify visualization of the PERMANOVA results, we show only filtered genera with a relative abundance ≥0.05.

Additionally, to assess the ecological processes shaping microbial community assembly by sampling location in the rhizosphere and root tissue, we calculated the β-nearest taxon index (βNTI). This was performed using the *iCAMP* package v.1.5.12 (Ning *et al*., 2020), following the approach described by Stegen *et al*. (2012) and recently utilized by Larsen *et al*. (2023). βNTI compares the observed phylogenetic turnover between communities with null expectations to infer the dominant ecological processes (e.g. dispersal limitation, homogeneous selection, variable selection or stochastic processes). Using boxplots and Wilcoxon tests, βNTI values were compared within and between sampling points, separately for each of the root-associated compartments (RS, RT), to evaluate the phylogenetic signature related to sampling point. A detailed description of the method is provided in the Supplementary Data as Supplementary information.

#### Genus-level associations with root anatomy and root architecture

We investigated associations between architectural and anatomical phenotypes and prokaryotic beta diversity using filtered (see section Taxon-level response to N and sampling location for details), rarefied and genus-aggregated datasets. First, we modelled medians of Bray–Curtis dissimilarities based on architectural and anatomical root phenotypes (Legendre and Anderson, 1999). This was done using constrained distance-based redundancy analyses (dbRDAs), implemented with the *dbrda* function from the vegan package. We performed these analyses separately for root architecture and root anatomy. Anatomy was analysed only in reference to microbiome data collected from crown roots at 0–20 cm depth. Depth was included in the models as a nesting factor for each root architectural phenotype. The resulting dbRDA models were then subjected to variable selection using the *ordistep* function from *vegan*. The selected variables were further analysed as predictors of genus median abundances in a model tested with PERMANOVA, using N as strata (via the *adonis2* function in *vegan*). Additionally, we calculated Spearman rank correlations between the median relative abundances and the root phenotype values found significant by PERMANOVA. These correlations were computed on the 100-fold subsampled datasets using the *cor.test* function from the *stats* package (R Core Team) and an FDR *P*-value correction with *q*-values (see the Results section for the exact *q*-values used for each trait).

## RESULTS

### N distribution in the mesocosms

The soil mixtures in combination with the N provided in fertigation provided different N availabilities in the potting media of our experiment. In the agricultural soil mixture, we found no significant differences in the mineral or total N content of the potting media between the two N treatments, nor was the vertical distribution of N significantly different across depths at harvest (Supplementary Data Fig. S2). Conversely, in the grassland soil mixture, the vertical distribution of mineral N in the potting media at harvest indicated nitrate mobilization to deeper soil layers. Its concentration tended to increase with depth, particularly under HN (Supplementary Data Table S2, Supplementary Data Fig. S2; Tukey HSD, HN: 0–20 vs 20–70 cm *P* = 0.043; 0–20 vs 70–150 cm *P* = 0.058). However, a significant reduction of nitrate below 70 cm for the grassland soil mixture under LN, compared with HN (Supplementary Data Fig. S2), indicates increased plant uptake in these layers under LN. Furthermore, depletion of total N from the deeper parts of the column in the grassland soil mixture under HN (Supplementary Data Fig. S2) points to plant uptake of N reserves. Ammonia and total carbon content did not differ across depths or N levels for the grassland soil mixture, indicating that plants primarily took up mineral N from fertigation.

### Shoot response to N treatment

The combination of N fertilization and two soil mixtures resulted in three distinct levels of plant growth, reflected by differences in shoot dry weight (Supplementary Data Table S3). We categorized these as three levels of N availability. The highest plant growth (averaging 9.6 g shoot dry weight, representing 100 % of the maximum growth in our system) was achieved with both the HN and LN treatments in the agricultural soil mixture, with no significant difference between the two N levels. This was followed by the HN treatment in the grassland soil mixture (6.8 g, a 25 % reduction in shoot dry weight compared with the maximum). Lastly, the LN treatment in the grassland soil mixture caused severe N deficiency, resulting in the lowest growth (1.67 g, an 80 % shoot dry weight reduction compared with the maximum value) (Supplementary Data Table S3). Specifically, shoot dry biomass, SPAD, total N content and chlorophyll content were approximately three times higher in the HN treatment compared with the LN treatment in the grassland soil mixture (Supplementary Data Table S3). Notably, no differences in shoot growth were observed between the N treatments in the agricultural soil mixture.

### Root response to the soil mixtures and N treatments

#### Architecture

**Fig. 2.**
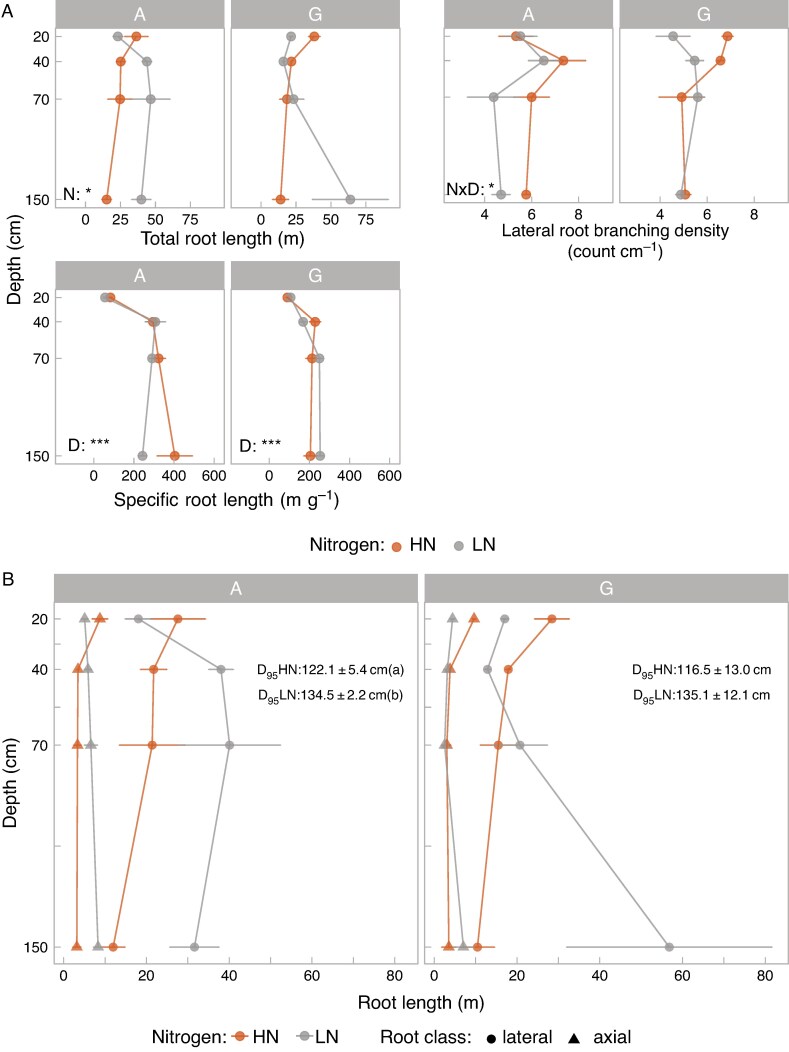
Root architecture in the mesocosms. (A) Root architectural phenotypes of plants in the two soil mixtures and under the two N levels across the different depths. A, agricultural soil mixture; G, Grassland soil mixture. Mean values and standard errors (*n* = 3) are shown. Significant ANOVA results of the factors N and depth are summarized for each subplot, corresponding to each soil mixture, as *P*-values: <0.05 (*); <0.0005 (***). The absence of *P*-values indicates no significant effect of N or depth. Extended ANOVA results are shown in Supplementary Data Table S3. (B) Root length distribution across mesocosm depths by root class (lateral and axial roots). Lateral root length, axial root length and D_95_ (depth above which 95 % of the roots are allocated) across the different combinations of soils with N treatment. Mean values and standard errors (*n* = 3) are shown. Different letters in the parentheses next to the D_95_ values indicate significant differences in this metric between N treatments for each soil mixture (*P*-value < 0.05).

We observed increased root allocation to deeper soil layers under LN in the two soil mixtures. Specifically, for the agricultural soil mixture under LN we saw significant increases across all depths: 38 % in total root length (TRL), 51 % in lateral root length (LRL) and 55 % in axial root length (ARL) (Supplementary Data Table S4, Fig. 2A). Additionally, roots allocated 16 % longer roots to deeper soil layers in the agricultural soil mixture under LN compared with HN (Fig. 2B). In the grassland mixture, rooting depth (D95, Fig. 2B), TRL and LRL showed similar trends to the agricultural soil mixture, without being significantly different between N treatments (Supplementary Data Table S4, Fig. 2B). Specific root length (SRL), which is directly related to the proportion of fine roots, significantly increased with depth in both soil mixtures. The only exception was the agricultural soil mixture under LN, where SRL decreased with depth (Supplementary Data Table S4, Fig. 2A). Regarding LRBD, we found no significant effects of N treatment in the agricultural mixture. However, LRBD tended to be greater in the shallow layers of the HN treatment in the grassland mixture. Lastly, no significant changes in the number of crown roots (e.g. axial roots) were observed between N treatments or soil mixtures (data not shown).

#### Anatomy

Root cross-section area (RXSA), living cortical area (LCA), total cortical area (TCA) and total stele area (TSA) were all around 70 % greater under HN compared with the LN treatment in the grassland soil mixture (Supplementary Data Table S5). Conversely, in the agricultural soil mixture, roots were significantly thicker under LN compared with HN, with RXSA increasing by 8 % (*P* = 0.016) and stele area increasing by 6 % (*P* = 0.032). However, neither LCA nor TSA showed significant differences between the N treatments in the agricultural soil mixture. Aerenchyma area (AA), percentage of cortical aerenchyma area (RCA) and percentage of living cortical area (pLCA) did not differ significantly between N treatments in either soil mixture.

### Microbial diversity across soil mixtures, N treatments, soil and root compartments and sampling locations

Observed richness (e.g. ASV counts) and the Shannon index decreased sequentially from bulk soil to rhizosphere to root tissue. Both metrics were significantly higher in the grassland soil mixture compared with the agricultural soil mixture. Conversely, differences in alpha diversity among N treatments were small and, overall, not statistically significant (Supplementary Data Fig. S3, Supplementary Data Table S6).

**Fig. 3.**
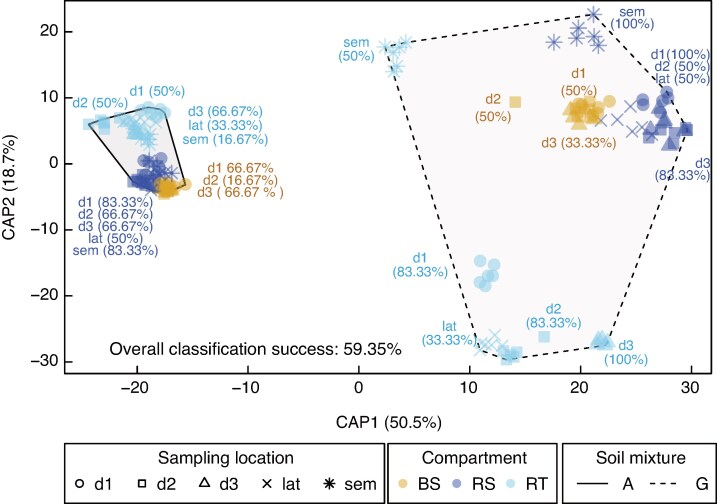
Separation of root prokaryotic community structure by the different experimental factors using constrained (canonical analysis of principal coordinates, CAP) multivariate analyses. Differences in prokaryotic communities constrained by the interaction between soil mixture (grassland or agricultural, by polygon outline), compartment (bulk soil, rhizosphere and root tissue, by colour) and sampling location (d1, d2, d3, lat, sem, by symbol shape). The amount of between-group variation of each CAP axis is provided in parentheses at each axis, and the CAP reclassification rates (%) for each soil mixture × compartment × sampling location are provided near the respective points, colour-coded by compartment.

Soil mixture, compartment and their interaction explained the largest proportion of variance in beta diversity (39, 12 and 10 % respectively; Table 2, Supplementary Data Fig. S4), with sampling location and N treatment accounting for 4.0 and 1.0 %, respectively. N effects on beta diversity were stronger in the grassland soil mixture compared with the agricultural soil mixture (Table 2). The rhizosphere and root tissue communities from plants grown in the grassland soil mixture exhibited better separation and higher dispersion by sampling location than those in the agricultural soil mixture (Fig. 3, Supplementary Data Fig. S4). This is further evidenced by the degree of discrimination in community structures among different treatments and sampling locations (Fig. 3). Specifically, the grassland soil mixture yielded the highest reclassification rates in the root tissue and rhizosphere compartments (50–100 %), in contrast to the agricultural soil mixture (16–83 %). The exception was the lateral root sampling location, which showed notably lower reclassification rates in both mixtures and compartments (16–50 %). This increased dispersion might be attributed to the wider depth range (0–70 cm) over which lateral roots were sampled, compared with other locations. Furthermore, the dispersion test of the entire dataset indicated that the observed differences in beta diversity across sampling locations and compartments in both soil mixtures may also have been driven by data heterogeneities between the levels of these two factors (see PERMDISP results in Table 2).

**Table 2.** Effects of soil mixture, N, compartment and sampling location on beta prokaryotic diversity calculated with multivariate permutational analysis of variance (PERMANOVA; degrees of freedom for each factor and error term are given in parentheses), calculated from Bray–Curtis dissimilarities based on relative ASV abundances. Main factors are soil mixture (grassland mixture, agricultural mixture), N treatment (high, low), compartment (bulk soil, rhizosphere, root tissue) and sampling location (for rhizosphere and root tissue samples: seminal roots, lateral roots, roots 0–20 cm, roots 20–70 cm, 70–150 cm; for bulk soil: 0–20 cm, 20–70 cm, 70–150 cm). Values represent the pseudo-*F* ratio (*F*), the permutation-based level of significance (*P*) and *R*^2^. *P* < 0.05 are bold.

Factor	PERMANOVA		PERMDISP		
	*F*	*R* ^2^	*P*	*F*	*P*
*Complete dataset*					
Soil mixture (*F*_1,154_)	**182**.**4**	**0**.**39**	**0**.**001**	**4**.**09**	**0**.**039**
N (*F*_1,154_)	**3**.**8**	**0**.**01**	**0**.**01**	0.84	0.363
Compartment (*F*_2,154_)	**27**.**7**	**0**.**12**	**0**.**001**	**27**.**79**	**0**.**001**
Sampling location (*F*_7,154_)	**4**.**4**	**0**.**04**	**0**.**001**	**6**.**36**	**0**.**001**
Soil mixture × N (*F*_3,154_)	**3**.**5**	**0**.**01**	**0**.**013**	1.58	0.191
Soil mixture × compartment (*F*_15,154_)	**23**.**9**	**0**.**10**	**0**.**001**	**33**.**04**	**0**.**001**
N × compartment (*F*_5,154_)	1.6	0.01	0.1	**10**.**45**	**0**.**001**
Soil mixture × sampling location (*F*_15,154_)	**3**.**1**	**0**.**03**	**0**.**001**	**6**.**28**	**0**.**001**
N × sampling location (*F*_9,154_)	1.7	0.01	0.049	**2**.**34**	**0**.**008**
Compartment× sampling location (*F*_12,154_)	1.3	0.02	0.159	**2**.**43**	**0**.**011**
Soil mixture × N × compartment (*F*_11,154_)	1.5	0.01	0.119	**12**.**36**	**0**.**001**
Soil mixture × N × sampling location (*F*_19,154_)	1.6	0.01	0.058	**3**.**43**	**0**.**001**
Soil mixture × compartment × sampling location (*F*_25,154_)	1.2	0.01	0.231	**6**.**36**	**0**.**001**
N × compartment × sampling location (*F*_25,154_)	0.8	0.01	0.731	0.37	0.999
Soil mixture × N × compartment × sampling location (*F*_51,154_)	0.8	0.01	0.745	**2**.**82**	**0**.**001**
*Grassland soil mixture*					
N (*F*_1,76_)	**5**.**523**	**0**.**034**	**0**.**002**	0.89	0.345
Compartment (*F*_2,76_)	**30**.**084**	**0**.**373**	**0**.**001**	**61**.**22**	**0**.**001**
Sampling location (*F*_6,76_)	**3**.**565**	**0**.**133**	**0**.**001**	**8**.**81**	**0**.**001**
N × compartment (*F*_2,76_)	**2**.**299**	**0**.**029**	**0**.**013**	**19**.**30**	**0**.**001**
N × sampling location (*F*_6,76_)	**1**.**510**	**0**.**056**	**0**.**046**	**4**.**93**	**0**.**001**
Compartment × sampling location (*F*_4,76_)	1.511	0.037	0.081	**11**.**17**	**0**.**001**
N × compartment × sampling location (*F*_4,76_)	0.890	0.022	0.625	**3**.**72**	**0**.**001**
*Agricultural soil mixture*					
N (*F*_1,76_)	1.893	0.015	0.068	0.128	0.718
Compartment (*F*_2,76_)	**22**.**514**	**0**.**350**	**0**.**001**	**28**.**48**	**0**.**001**
Sampling location (*F*_6,76_)	**2**.**243**	**0**.**105**	**0**.**002**	**4**.**924**	**0**.**001**
N × compartment (*F*_2,76_)	0.778	0.012	0.686	**10**.**2**	**0**.**001**
N × sampling location (*F*_6,76_)	1.104	0.052	0.292	**2**.**645**	**0**.**006**
Compartment × sampling location (*F*_4,76_)	1.109	0.034	0.293	**4**.**743**	**0**.**002**
N × compartment × sampling location (*F*_4,76_)	0.908	0.028	0.567	**2**.**544**	**0**.**003**

**Fig. 4.**
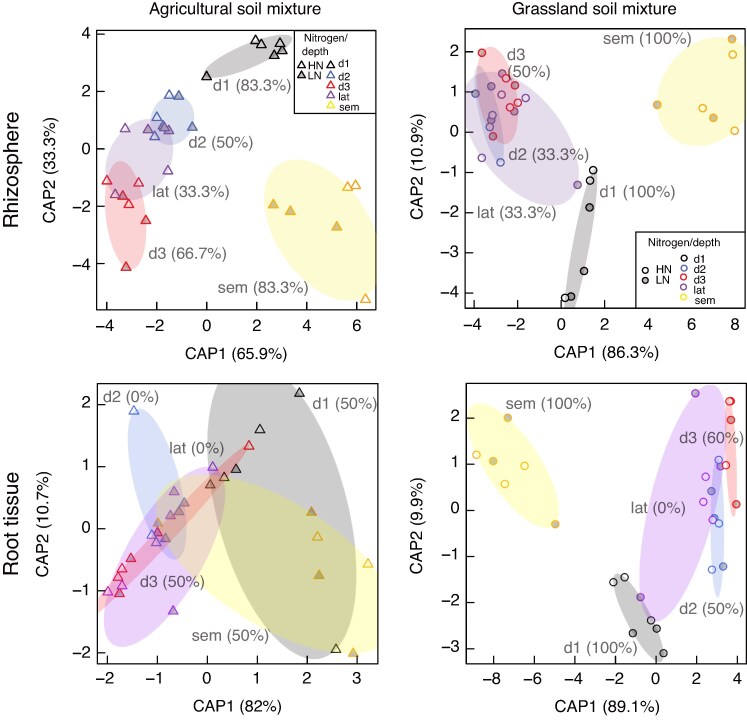
Separation of root prokaryotic community structure by sampling location, using canonical analysis of principal coordinates (CAP) on Bray–Curtis dissimilarities, calculated from relative ASV abundances. Plots were made separately by compartment (rhizosphere and root tissue) and soil mixture (agricultural and grassland). Points filled by N level: H (high) or L (low). Sampling locations: crown roots 0–20 cm (d1), crown roots 20–70 cm (d2), crown roots 70–150 cm (d3); seminal roots (sem), lateral roots (lat). A visualization of the different sampling locations is shown in Fig. 1.

When analysed by soil mixture and compartment, we observed a clear separation of the seminal prokaryotic communities from the crown root communities above 20 cm and the rest of the sampling locations (Fig. 4). This indicated a switch in biodiversity at 20 cm within the rhizosphere of both soil mixtures, regardless of the N treatment. The root tissue communities of the agricultural soil mixture, however, showed a less defined separation based on sampling locations. A detailed analysis of genus-level diversity by soil mixture and compartment is presented below.

### General distribution of ASVs across compartments, soil mixtures and N treatments

The complete list of all 19 974 detected bacterial and archaeal taxa, summarized from phylum to ASV, is provided in Supplementary Data File S2, and visualized by phyla and genera in Supplementary Data Fig. S5. The percentages (and ASV counts) of the total ASVs that were classified to the different taxonomic ranks were 99.6 % (19 891, phylum), 98.4 % (19 652, class), 92.1.7 % (18 405, order), 77.8 % (15 545, family) and 42.0 % (8390, genus). Overall, 49 phyla, 111 classes, 239 orders, 318 families, 708 genera and 260 species were identified. Differences in the distribution of taxa at the phylum level correspond to the analyses of beta diversity, with differential patterns by soil mixture and compartment (Supplementary Data Fig. S5). After applying filters for rare, sparse and outlier ASVs we obtained a total of 9635 ASVs.

The indicator species statistics for the filtered 9635 ASVs by N level and soil mixture are provided in the Supplementary Data File S3 (visualized in a Venn diagram in Supplementary Data Fig. S6A). Notably, 20 and 63 ASVs were significantly associated with LN and HN, respectively, accounting for the two soil mixtures and all compartments. Among the most abundant ASVs under LN common in the two soil mixtures we found the genera *Streptomyces* and *Variovorax* and unclassified genera from the families *Sandaracinaceae* and *Chitinophagaceae* (Supplementary Data File S3). *Stenotrophomonas*, *Achromobacter*, *Roseateles* and unclassified genera of *Enterobacteriaceae* were enriched under HN. A similar analysis applied by compartment and N showed that root tissue had greater specific ASVs, sharing only >200 ASVs with rhizosphere (Supplementary Data Fig. S6B). Among the most abundant ASVs, indicators of rhizosphere under LN, we found some from the genera *Stenotrophobium* (Pseudomonadota), *RS25G* (Verrucomicrobia) and *Occallatibacter* (Acidobacteriota) (Supplementary Data File S4). Together, the indicator ASVs under these conditions covered a total of 31 genera. Among the indicator ASVs of root tissue under LN we found the genera *Streptomyces* (Actinomycetota), *Burkholderia–Caballeronia–Paraburkholderia* and *Dyella* (Pseudomonadota), and together the indicator ASVs covered 76 genera (Supplementary Data File S4). Interestingly, ASVs from the genus *Sphingomonas* were found indicative of both rhizosphere and root tissue. A detailed description of N and sampling location-sensitive genera by compartment is presented below.

### N-sensitive genera by soil mixture and compartment

#### Rhizosphere

**Fig. 5.**
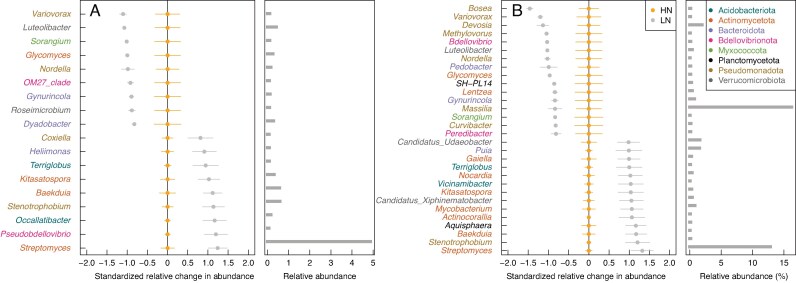
N-sensitive genera in the rhizosphere (A) and root tissue (B) of plants growing in the grassland mixture found with permutational analyses of variance (PERMANOVA) of each genus. Genera with *q* ≤ 0.05, *F* ≥ 5 and relative abundance ≥0.05 for N are shown. Genus names are colour-coded by phyla. The standardized relative mean difference between LN and HN and standard errors are provided for each taxon. Relative abundance per genus, in reference to the subset dataset by soil mixture and compartment, is shown on the right.

In the rhizosphere of the grassland mixture mesocosms, the most abundant genera under LN conditions included *Streptomyces*, *Nocardia* and *Baekduia* (Actinomycetota), *Stenotrophobium* (Pseudomonadota) and *Peribacillus* (Bacillota) (Fig. 5A). No N-sensitive genera were identified in the rhizosphere of the agricultural soil mixture columns.

Additionally, we investigated the presence of ammonia-oxidizing bacteria and archaea to assess potential microbial competition for N with the plant in the rhizosphere (Qin *et al*., 2024) (Supplementary Data Fig. S7). The bacterial genera *Nitrosomonas* and *Nitrosospira*, along with the archaeal genus *Nitrosarchaeum*, tended to increase in the rhizosphere of plants growing under LN in the grassland soil mixture. The bacterial genus *Nitrosococcus* and the archaeal genus *Nitrosarchaeum* exhibited a similar trend in the rhizosphere of the agricultural soil mixture. However, none of these changes were statistically significant between N levels or sampling locations. In no instance did their relative abundance exceed 0.4 % within their respective subset datasets (subsets by compartment and soil mixture). These results suggest that microbial competition for ammonia in the rhizosphere of our experimental system is limited.

#### Root tissue

Among the most abundant genera enriched under LN in the root tissue of the grassland soil mixture, we found genera from the phyla *Streptomyces* and *Mycobacterium* (Actinomycetota), *Puia* (Bacteroidota), and *Candidatus* Udaeobacter (Verrucomicrobiota) (Fig. 5B). Although *Streptomyces* was enriched under LN in both the rhizosphere and root tissue, its relative abundance was almost three times higher in the root tissue. The genus *Massilia* was enriched with the highest relative abundance under HN in the root tissue of the grassland soil mixture. Consistent with the rhizosphere, no N-sensitive genera were identified in the root tissue of the agricultural soil mixture columns.

When examining ammonia-oxidizing bacteria and archaea in root tissue, we found results similar to those in the rhizosphere: no significant effect of N or sampling location on the abundance of the targeted genera was observed (Supplementary Data File S1).

### Sampling location-sensitive taxa

#### Rhizosphere

**Fig. 6.**
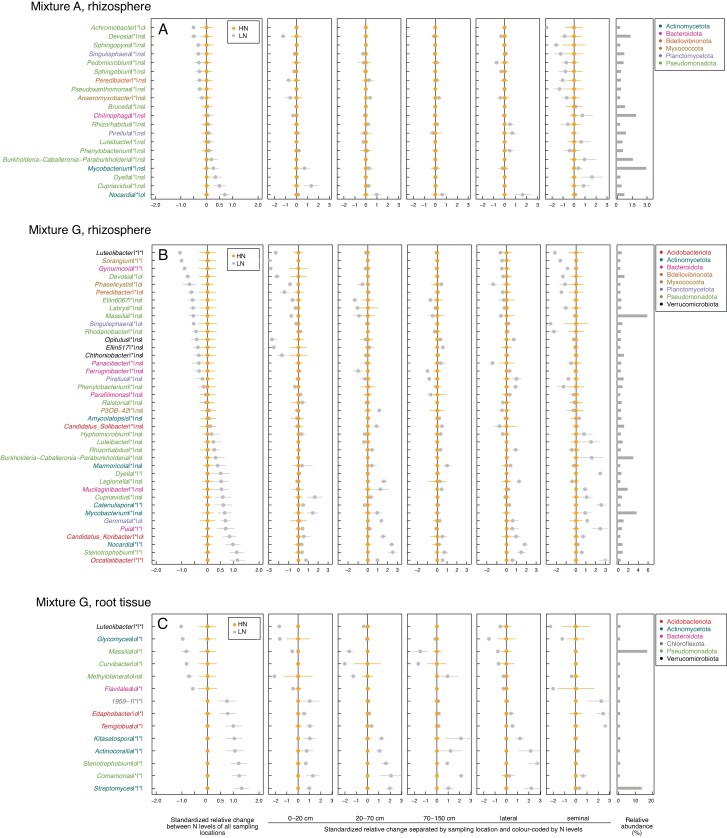
Sampling point-sensitive genera shown in separate panels by compartment and sampling location. Genera found with individual permutational analyses of variance (PERMANOVA) per genera, where both N and sampling location (and their interaction) were included in the models. Genera with *q* ≤ 0.05, *F* ≥ 5 (for the factor sampling location) and relative abundance ≥0.05 are shown. The standardized relative mean and standard errors are provided for each genera. Genus names are colour-coded by phyla. The significance of the PERMANOVA test is indicated after each genus name, between vertical lines: the first argument represents the significance of sampling location, the second the significance of N: *q ≤ 0.05; o*, q* ≤ 0.1; ns, not significant. The relative abundance in percentage per genus is given from the total of each subset of soil mixture and compartment as horizontal bars on right. Note that sampling location was not significant to the root tissue microbial diversity in the agricultural soil mixture (A), and therefore its panel is not shown. G, grassland soil mixture.

In the agricultural soil mixture (Fig. 6A) most differences in relative abundance along the maize root system were observed in the seminal roots for both HN and LN. *Dyella*, *Paraburkholderia* (Pseudomonadota), *Chitinofaga* (Bacteroidota) were enriched in the seminal roots under LN, while other genera, such as *Pseudoxanthomonas*, *Sphingopyxis* and *Sphingobium* (Pseudomonadota) were depleted under LN. In the grassland soil mixture (Fig. 6B) the genera *Luteolibacter* (Verrucomicrobiota), *Sorangium* (Myxococcota), *Gynurincola* (Bacteroidota), *Devosia* (Pseudomonadota), *Opitutus* and *Ellin517* (Verrucomicrobiota) were enriched in the crown and seminal roots above 20 cm under HN, and the genus *Copriavidus* (Pseudomonadota) was enriched below 20 cm and HN. *Legionella* (Pseudomonadota), *Nocardia* and *Stenotrophobium* (Actinomycetota) were enriched in the mid part of the columns and in the lateral roots (20–70 cm) under LN.

#### Root tissue

In the grassland soil mixture (Fig. 6C) the seminal root displayed a contrasting pattern in terms of relative abundance of specific taxa, when compared with the other sampling locations. While genera such as *Streptomyces* and *Kitasatospora* (Actinomycetota) were enriched in most sampling locations under LN, they did not change with N in the seminal root. Conversely, *Massilia* (Pseudomonadota) was depleted under LN but also showed no change with N in the seminal root. No specific sampling-point-sensitive taxa were found (with *q* < 0.05) in the root tissue of plants growing in the agricultural soil mixture.

### Ecological community assembly and phylogenetic signature at different sampling locations

We initially observed median βNTI values greater than +2 for both within and between comparisons across all rhizosphere sampling locations, encompassing both soil mixtures and N levels (Wilcoxon test, *P* > 0.05; Supplementary Data Fig. S8A). This indicates phylogenetic overdispersion at the different sampling locations, meaning prokaryotic species within or between these communities are more distantly related than would be expected by chance. This pattern serves as a signature of deterministic processes, such as variable selection or dispersal limitation, acting as the main assembly drivers for microbial communities across the sampling locations in the rhizosphere (Stegen *et al*., 2012; Larsen *et al*., 2023).

However, when similar analyses were performed on datasets split by soil mixture and compartment (Supplementary Data Fig. S9A, B), median βNTI values were generally smaller than with the complete dataset, specifically falling between −2 and +2. This shift indicates a phylogenetic signature predominantly influenced by stochastic processes at the different sampling locations of both rhizosphere and root tissue. This reduction in βNTI values, observed when the datasets were analysed within specific soil mixtures (thereby controlling for the soil effect on βNTI), suggests that soil mixture is the primary determinant of differences in microbial community assembly within the rhizosphere.

For root tissue communities, initial βNTI median values were generally smaller than those observed in the rhizosphere (Supplementary Data Fig. S8B). Notably, in the combined root tissue analysis, only the median βNTI value for within comparisons was greater than +2, indicating a tendency towards more deterministic assembly and phylogenetic overdispersion for communities within a given sampling location than between different sampling locations. Similar to the rhizosphere, but to a lesser extent, root tissue βNTI values decreased when controlling for soil mixture (Supplementary Data Fig. S9C, D), falling into the range indicative of stochastic phylogenetic assembly.

Overall, these results demonstrate that the ecological processes determining root microbial diversity are highly soil-specific. When the confounding effect of soil mixture is accounted for by analysing subsets of the data, the assembly of root microbial communities tends to be driven more by stochasticity. Conversely, the significant deterministic signals observed in the overall dataset are largely attributable to the selective pressures or dispersal limitations imposed by the different soil mixtures.

### Correlations between root anatomy or architecture and beta diversity

In general, anatomical phenotypes correlated with beta diversity, separating the communities by N fertilization (Supplementary Data Fig. S10A, B), while architectural phenotypes correlated with separation by sampling depth as shown by the dbRDA (Supplementary Data Fig. S10C, D). However, our PERMANOVA results indicated that the anatomical phenotype root-cross section area (RXSA) was only marginally significant to the rhizosphere beta diversity (*P* = 0.083, *F*_1,5_ = 1.65; *R*^2^ = 0.29) of the grassland mixture. None of the anatomical phenotypes were significant to the beta diversity in the root tissue in this mixture, nor in either root compartment of the agricultural soil mixture. Among the architectural phenotypes, LRBD was significant to the rhizosphere and the root tissue beta diversity in the grassland soil mixture. LRBD was also marginally significant to the rhizosphere beta diversity in the agricultural soil (Table 3). Furthermore, in the grassland soil mixture the architectural phenotypes TRL (total root length), LRL (lateral root length) and LRBD collectively explained ∼30 % of the variance in rhizosphere beta diversity. In contrast, only LRBD was significant to beta diversity of the root tissue in this soil mixture (Table 3). Based on these results, we further searched for significant correlations between the relative abundance of the different genera with the architectural phenotypes in the plants growing in the grassland soil mixture.

**Table 3.** Effects of architectural root phenotypes on the prokaryotic beta diversity. The effect of the variables was quantified by PERMANOVA on Bray–Curtis dissimilarities. LRBD was selected for the agricultural rhizosphere soil and LRBD, TRL and LRL for the grassland soil using stepwise selection models. Significant values with *P* > 0.05 are bolded.

Factor	Grassland soil mixture			Agricultural soil mixture		
	*F*	*R* ^2^	*P*	*F*	*R* ^2^	*P*
*Rhizosphere*						
**TRL**	**1**.**7**	**0**.**09**	**0**.**018**	–	–	–
**LRL**	**1**.**6**	**0**.**08**	**0**.**045**	–	–	–
**LRBD**	**2**.**1**	**0**.**11**	**0**.**003**	1.4	0.08	0.065
Residual		0.72			0.92	
Total		1.00			1.00	
*Root tissue*						
TRL	1.3	0.07	0.143	0.6	0.04	0.849
LRL	1.6	0.09	0.314	0.9	0.05	0.418
**LRBD**	**2**.**0**	**0**.**11**	**0**.**005**	0.9	0.06	0.409
Residual		0.73			0.85	
Total		1.00			1.00	

### Correlations between root architecture and relative abundance of prokaryotic genera

At the genus level, we found 14 genera that were highly correlated with LRBD in the rhizosphere of the grassland mixture. From these genera, the relative abundance of *Ferruginibacter* was positively correlated with LRBD. For other genera, including *Mucilaginibacter*, *Nocardia*, *Pseudomonas* and *Stenotrophobium*, abundances were negatively correlated with LRBD (Supplementary Data Fig. S11). Total root length (TLR) was also significant to the rhizosphere beta diversity in the grassland soil mixture, with 11 genera. *Rhizobium*, *SH-PL14* and *Tahibacter* showed negative correlations, while *Gaiella*, *Candidatus* Udaeobacter and *Candidatus* Alysiosphaera showed positive correlations with TRL (Supplementary Data Fig. S12).

Significant correlations between LRBD and prokaryotic genus abundance were weaker in the root tissue compared with the rhizosphere, as indicated by higher *q*-values of the FDR (Supplementary Data Fig. S13). Eleven genera were correlated with LRBD in the root tissue. Among these, *Massilia*, *Rickettsia* and *Rhizobacter* had increased abundances with increased LRBD. The other genera, such as *Dokdonella*, *Rudaea* and *Peredibacter*, decreased, while *Wolbachia* exhibited an unclear trend (Supplementary Data Fig. S13).

## DISCUSSION

### Relevance of this study for microbiome under limiting N supply

In this study, we dissected the maize rhizosphere and root microbiome of a 4-week-old (phenological stage V5–V7) inbred genotype. We grew plants under two N treatments in combination with two soil mixtures, using large 30-L, 1.5-m tall mesocosms. Our aim was to identify differences in prokaryotic communities at the centimetre scale, with sampling locations distributed across three depths (0–150 cm), two root origins (seminal vs crown) and fine roots (lateral roots only). We differentiated the soil–plant compartments bulk soil, rhizosphere and root tissue. We did not focus on root tips since longitudinal gradients of root microbes near them are already well documented (Rüger *et al*., 2021; Yu *et al*., 2021; Wang *et al*., 2024). Interactions between V5–V7 maize root architecture and microbes cannot be reliably studied at the centimetre scale in small containers, as roots grow too large, resulting in an unrealistic distribution compared with field conditions. In our study, roots indeed reached the bottom of the 1.5-m columns by harvest. These mesocosms have the potential to include soil gradients, allowing us to test hypotheses about interactions between the root microbiome and root phenotypes within a vertical, dynamic matrix. To our knowledge, this study is pioneering in describing the spatial distribution of microbes under LN conditions in a soil volume that more closely resembles the available space that a 4-week-old plant explores in the field (Galindo-Castañeda *et al*., 2018). We aim to close the gap in our knowledge about the interactions between adaptive root phenotypes and root microbiomes by providing a larger volume of soil where microbes are studied at a developmental plant stage relevant for N acquisition.

The LN treatment in the agricultural soil mixture was not associated with reduced plant growth; instead, it triggered deeper rooting compared with HN (Supplementary Data Fig. S2). This indicates a root response to the positive N gradient with depth, which successfully nourished the plant to optimal growth (Supplementary Data Table S3). Interestingly, these changes in rooting depth under varying fertilization were not associated with changes in the prokaryotic beta diversity of the root tissue or rhizosphere in the agricultural soil mixture (Table 3). This suggests that, under moderate N limitation, roots may respond more independently of microbes in this specific soil context. In contrast, the N treatment was a significant driver of beta diversity in the grassland soil mixture (Table 3). Furthermore, we identified N-responsive taxa in this soil mixture. The enhanced response of root-associated taxa under limiting N conditions has been documented for maize (Mukhtar *et al*., 2025) and points to a possible stress-specific interdependence between the plant and its associated microbes. Here, we demonstrate that this response is also specific to the depth and location along the root, as shown by the significant interaction of N and compartment, and N and sampling location in the grassland soil mixture (Table 3). This supports the hypothesis that root architecture structures root–microbiome associations under LN conditions.

### Horizontal differentiation: mesocosms allow soil–root differentiation between bulk soil and root tissue

Our mesocosm system successfully replicated several known aspects of maize root microbiome biology. Consistent with previous maize microbiome surveys (Peiffer *et al*., 2013; Walters *et al*., 2018; Jeewani *et al*., 2020), we found that the soil mixture had the primary effect on the microbiome, followed by the compartment. We observed a significant decrease in alpha diversity moving from the bulk soil to the rhizosphere and then to the root tissue (Supplementary Data Fig. S3). This pattern is well established in maize (Jeewani *et al*., 2020; Wang *et al*., 2024). Particularly in our experimental system, we observed a clear differentiation of the communities between bulk soil and rhizosphere in each pot. This compartmentalization within the same pot reduces the need for unplanted pots to study bulk soil biodiversity, which can introduce biases. Bulk soil in unplanted pots might differ from bulk soil influenced by resource gradients created by plant growth (Zhang *et al*., 2021). In our study, the compartment significantly influenced both alpha diversity (explaining ∼70 % of the variance) and beta diversity (explaining 11–13 % of the variance), aligning with the aforementioned studies.

### Compartment and N-responsive prokaryotic genera

Our indicator species analyses of compartment- and N-responsive taxa revealed several genera that might play a key role in the interactions between maize roots and their associated microbiome under LN (Fig. 5, Supplementary Data Fig. S6). For instance, in the root tissue, there were double the number of genera corresponding to indicator ASVs (76) compared with the rhizosphere (31) (Supplementary Data Fig. S6), showing the selective specificity of the root environment. Notably, the root tissue communities from the two soil mixtures were also more similar in the unconstrained ordination plots (Supplementary Data Fig. S3) compared with the rhizosphere communities, demonstrating the strong plant control on the prokaryotic colonization of the root tissue. Specifically, the genera *Streptomyces* and *Massilia* were notably abundant in the root tissue of the grassland soil mixture, and these two genera were also indicative of this compartment (Supplementary Data File S4). *Streptomyces* was more abundant under LN and *Massilia* more abundant under HN in the grassland soil mixture (Fig. 5). *Streptomyces* is well known for its plant growth-promoting phenotypes, such as producing indolic compounds, siderophores, ACC deaminase and phenazines (Nozari *et al*., 2021), improving maize tolerance to abiotic stress.

Our findings regarding *Massilia* and maize roots align well with recent research, further solidifying this genus’s potential importance in the rhizosphere and root microbiome. First, we observed *Massilia* to be highly abundant within the root tissue. This echoes findings from a large-scale maize root microbiome study, which identified strains from this genus as keystone species (He *et al*., 2024). Our results also coincide with those of Wang *et al*. (2024), who reported greater abundance of *Massilia* in the maize root cortex compared with the rhizosphere. Second, our study provides insights into *Massilia*’s response to N levels. Yu *et al*. (2021) suggested that the abundance of *Massilia* in the rhizosphere might be enhanced by plant flavones under LN conditions. Our data support this, as we found *Massilia* to be enriched under HN conditions in the grassland soil mixture, where plants were still experiencing mild N limitation (Supplementary Data Table S2). Conversely, *Massilia* was reduced under LN in the same soil mixture. This reduction under severe N limitation, when fixed carbon for exudates is extremely limited, could indicate that *Massilia* associations may become less sustainable for the plant’s carbon/nitrogen economy. Third, we observed a positive correlation between *Massilia*’s endospheric relative abundance and LRBD (Supplementary Data Fig. S13). This observation has also been reported in previous inoculation experiments (Yu *et al*., 2021; Wang *et al*., 2024), reinforcing the potential role of *Massilia* in influencing root architecture.

In the root tissue, ASVs from an unidentified genus of the order *Saccharimonadales* was found in high abundance (Supplementary Data File S4). This order has been reported to increase with sugars added to soils where maize is grown, and it has been proposed to participate in phosphorus cycling by influencing alkaline phosphatases in the maize rhizosphere (G. Wang *et al*., 2022). Other highly abundant ASVs indicative of root tissue and rhizosphere under LN belonged to the genus *Sphingomonas*, which has been reported as a plant growth-promoting bacterium (PGPB) in inoculation experiments with maize (Molina-Romero *et al*., 2017; F. Wang *et al*., 2022). Its high abundance across the rhizosphere and root tissue (Supplementary Data File S4) might indicate either permeability through the root cortex under severe LN limitation, or persistence from the seed. *Sphingomonas* might be an interesting genus to be further explored in inoculation experiments in maize due to its persistence outside and inside the root under LN conditions.

### Vertical differentiation: depth and sampling location are key factors in assembly of root microbial communities

Our findings highlight that sampling location significantly influences beta diversity (Table 2) within the root microbial communities. Further analysis, by splitting datasets based on soil mixture and compartment, revealed that the seminal root system harbours a unique microbiome, distinctly separated from other sampling points (Figs 3 and 4). This aligns with previous research on *Lolium perenne* (Tannenbaum *et al*., 2020) and wheat (Quiza *et al*., 2023), which suggests that the seminal root system provides different microbial niches compared with the mature root system. The origin of the seminal microbiome could be the seed, but also the inherent differences in functions between the seminal and the crown root systems (Hochholdinger, 2009). The former is more reliant on seed supply for nutrients during the first days, while the latter depends on external nutrient availability and provides anchorage to the growing plant. We also observed a clear separation between the crown root system above 20 cm and deeper roots. This is likely due to vertical soil gradients, such as reduced oxygen levels, decreased root density and, in some cases, a reduction in N availability (Supplementary Data Fig. S2). Based on these results, we conclude that sampling location is a critical consideration in maize root–microbiome analyses. Researchers must carefully consider whether to collect a truly representative sample of the root-associated microbiome or to minimize microbiome variability within mesocosm experiments.

### Ecological processes driving microbiome assembly along the maize root system.

Our study reveals that the primary factor driving the deterministic assembly of the root microbiome in both the rhizosphere and root tissue across different locations is the soil mixture, rather than the specific sampling location (Supplementary Data Figs S9 and S10). In our experiment, sampling locations influenced microbiome assembly in a more random fashion (Supplementary Data Fig. S10). This suggests that environmental filters imposed by each sampling location might be less important than other factors, like dispersion (which, in this context, could refer to the initial soil inoculum), ecological drift (growth dynamics of the microbial population), or priority effects (which might be significant in a system containing 5–15 % soil) (Li and Gao, 2023). In the context of microbiome selection using plants with different root architectures, this result implies that the composition of the root microbiome is hard to predict using solely the root locations that we used here at this stage in plant growth.

These results partially align with a previous study on maize root microbiome assembly under optimal N supply (Rüger *et al*., 2021). That study found deterministic processes were more dominant in mature root tissue zones, while stochastic processes prevailed in root tips. Since our study did not perform a fine-scale differentiation between root tips and mature root zones, our results likely combine young and mature root zones. This could make stochastic processes appear more relevant given the large number of root tips found in our samples. Other research has also shown the importance of stochastic processes in root microbiome assembly (Attia *et al*., 2022; Li and Gao, 2023).

### Correlations between root phenotypes and root microbial diversity

In this single-genotype experiment, we were able to find significant correlations between root architectural phenotypes and the relative abundance of some taxa in a soil mixture-dependent fashion. Specifically, LRBD, LRL, and TRL together explained ∼30 % of the beta diversity in the grassland soil mixture, where we had reduced N input (Table 3). However, the same root phenotypes were not significant in the agricultural soil mixture, where we had optimal N supply. These results suggest that the interaction between microbes and root phenotypes might be more crucial under LN conditions, and that this interaction is also soil-dependent. Furthermore, observing these correlations within a single maize genotype points to the possibility that intragenotype plasticity in root phenotypes could be relevant for shaping microbial associations. Despite finding significant correlations, the causal relationship between microbes and root phenotypes remains to be fully explored. Are these correlations driven by the plant, by the microbes, or by another mechanism not directly related to their interaction? These are compelling open questions for future research.

Our findings reveal a complex and often counter-intuitive relationship between maize root architecture and its associated microbial communities, particularly under limiting N conditions. In grassland soils, most of the correlations we observed between LRBD and the abundance of microbial genera in the rhizosphere were negative (Supplementary Data Fig. S11). This suggests that an increased LRBD might actually hinder the plant’s ability to sustain microbial growth. This observation challenges the common assumption that increased LRBD, often promoted by microbes, directly leads to enhanced nutrient uptake and plant growth (Hungria *et al*., 2022). In fact, root phenotyping studies on maize under N limitation indicate that reduced, rather than increased, lateral branching density is an adaptive trait (Lynch *et al*., 2023). Maize plants with long but fewer lateral roots are more efficient at acquiring N under limited supply (Zhan and Lynch, 2015). In this context, if bacteria are to genuinely promote plant growth under LN, strains that lead to an increase in LRBD could potentially be detrimental. This might be especially true under severe N scarcity, as the development of numerous lateral roots carries a high metabolic cost that can be critical when resources are scarce (Rangarajan *et al*., 2022). Plants might also adapt by limiting or altering the amount and composition of their root exudates when under LN with many lateral roots, aiming to reduce this metabolic drain. This, in turn, would likely decrease the abundance of associated bacteria. Conversely, increased lateral root length, which is adaptive under LN (Lynch *et al*., 2023), seems to promote microbial associations by showing mostly positive correlations with taxa abundance (Supplementary Data Fig. S12). If the plant can effectively acquire more N with longer and fewer lateral roots, it might still have some resources to sustain microbial associations using root exudates. These hypotheses merit further investigation.

Correlations between root tissue genus abundance and LRBD show diverse responses, probably determined by interactions in the root cortex (Supplementary Data Fig. S13). For example, bacteria from the genus *Massilia* had a positive correlation with LRBD, in accordance with previous findings that bacteria from this genus seem to be associated with increased LRBD (Wang *et al*., 2024). Interestingly, in this experiment, we did not find significant differences in most measured anatomical phenotypes, nor did we observe significant correlations between beta diversity and anatomy. While anatomical measurements were only taken at one spot in the root crown, potentially limiting our ability to find correlations, the lack of phenotypic response to our experimental conditions suggests stable anatomical expression and reduced plasticity within the genotype we used. Previous studies indicate that plasticity in the expression of anatomy varies in maize populations, with genotypes showing larger plasticity than others in their anatomies (Yang *et al*., 2019).

### Future research avenues

To help advance our understanding of the associations between root architecture and microbial genera, future research should focus on several key areas. First, it is crucial to study plants with more diverse root phenotypes, either from diverse genotypes or from plants with high root plasticity belonging to the same genotype or variety. This will allow us to observe how different root forms influence microbial communities. Second, investigating the metabolites involved in these interactions, and their location at different depths, across root classes and root types, would shed light on the specific mechanisms involved in the location specificity of microbe–root associations. Third, a deeper understanding of the genetic control of LRBD and lateral root length is essential. Such knowledge would facilitate the development of mutants with varying expression of these phenotypes. These mutants would, in turn, enable targeted experimental work with microbes, allowing validation of the correlations observed in studies like ours. However, while mutants offer valuable tools, deploying the natural variation of root phenotypes found within maize populations in microbiome studies will provide a more holistic understanding of microbial associations. Mutants can sometimes exhibit pleiotropic effects on other plant traits that might also be important for microbial interactions (Galindo-Castañeda *et al*., 2022). By examining natural variation, we can capture the full complexity of these relationships. Ultimately, by deciphering the mechanisms behind microbial associations with LRBD and LRL across diverse maize populations, we can work towards designing specific genotype–microbe combinations that effectively enhance maize N uptake, contributing to more sustainable agricultural practices.

### Conclusions

In this study, we have provided a detailed characterization of root-associated prokaryotic communities within a 1.5-m-deep mesocosm system under three levels of N limitation. Our work reveals that sampling location along the root system is a crucial, yet often overlooked, factor influencing the composition of both the rhizosphere and root tissue microbiome, especially when N is limited. It emerged as the third most important factor, after soil mixture and root compartment (rhizosphere, root tissue), strongly emphasizing the critical role of root architecture in shaping the root microbiome. Notably, we found that seminal root biodiversity is not representative of crown root biodiversity in 4-week-old maize plants. This is a key distinction largely missed by most large-scale microbiome surveys, which often do not account for such fine-scale spatial and architectural differences. Our findings therefore highlight the urgent need for researchers to carefully consider sampling location and root architectural variations in future maize root microbiome studies.

Aligned with previous research, we observed that stochastic phylogenetic assembly drives the differences in prokaryotic communities along the root system. Furthermore, specific root architectural phenotypes – lateral root branching density and lateral root length – were significantly correlated with the abundance of 37 prokaryotic genera under limiting N conditions, pointing to key interactions that warrant further mechanistic investigation. Overall, our results underscore the important, yet understudied, potential of harnessing root adaptations in combination with soil microbiomes to enhance N uptake efficiency for a more sustainable agriculture.

## Supplementary Material

mcaf185_Supplementary_Data

## Data Availability

The raw sequences have been deposited in the European Nucleotide Archive under accession number PRJEB88070.

## SUPPLEMENTARY DATA

Supplementary data are available at *Annals of Botany* online and consist of the following. Table S1: N content of the soils used in the two soil mixtures. Table S2: effects of N treatment and depth on N content of the two soil mixtures at harvest. Table S3: plant growth metrics across soil mixtures and N treatments. Table S4: effects of N and depth on architectural phenotypes calculated with ANOVA. Table S5: root anatomical phenotypes shown as mean ± standard deviation. Table S6: effects of soil mixture, N treatment, compartment and sampling location on alpha prokaryotic diversity. Figure S1: rarefaction curves by compartment and soil mixture. Figure S2: N and carbon content in the two soil mixtures by depth at plant harvest. Figure S3: alpha diversity per compartment. Figure S4: separation of root prokaryotic community structure by the different experimental factors using unconstrained (principal coordinate ordinations, PCoA) multivariate analyses on the median values of Bray–Curtis dissimilarities. Figure S5: relative abundance of major prokaryotic phyla (A) and genera (B) across the 155 samples. Figure S6: indicator species statistics by soil mixture and N levels, and across the N levels and compartments. Figure S7: ammonia-oxidizing genera, plus the nitrite-oxidizing bacterium *Nitrolancea*, and their relative change and relative abundance in the rhizosphere of the two soil mixtures. Figure S8: dominant community assembly processes and phylogenetic signatures by sampling location measured through the distribution of pairwise β-nearest taxon index (βNTI) of rhizosphere and root tissue Figure S9: dominant community assembly processes and phylogenetic signatures by sampling location measured through the distribution of pairwise β-nearest taxon index (βNTI) split by rhizosphere (panels A and B) and root tissue (panels C and D) for each soil mixture (grassland, G mixture (panels A, C) and agricultural (panels B, D)). Figure S10: distance-based redundancy analyses of anatomical (A, B) and architectural (C, D) root phenotypes as predictors of beta diversity based on Bray–Curtis dissimilarities of rhizosphere and root tissue. Figure S11: scatterplots of lateral root branching density vs relative abundance of genera in the rhizosphere of the grassland soil mixture. Figure S12: scatterplots of total root length vs relative abundance of genera of the rhizosphere in grassland soil mixture. Figure S13: scatterplots of lateral root branching density vs relative abundance of genera in the root tissue of the grassland soil mixture. Supplementary information: statistical analyses followed to measure ecological processes shaping microbial community assembly by sampling location in the rhizosphere and endosphere. File S1: PERMANOVA results at the ASV level for all filtered ASVs found in this study. File S2: taxonomical summary of all unfiltered ASVs. File S3: indicator species results for the model including soil mixture and N and the interaction of these two factors on filtered ASVs. File S4: indicator species results for the model including compartment and N and the interaction of these two factors on filtered ASVs. Supplementary Video: experimental system and sampling.
