# Supplementary material for: Locating the microbes along the maize root system under nitrogen limitation: a root phenotypic approach
Source: Ann Bot. 2025 Aug 12;136(5-6):1143–62. doi: 10.1093/aob/mcaf185 (PMC12682841; doi:10.1093/aob/mcaf185)
Supplement: mcaf185_Supplementary_Data [file mcaf185_supplementary_data.zip › Galindo_etal_supp_materials.pdf]

SUPPLEMENTARY MATERIAL

Title: Locating the microbes along the maize root system under nitrogen limitation: a root phenotypic approach

Galindo-Castañeda *et al.*, 2025

1. Supplementary tables

Table S1. Nitrogen content of the soils used in the two soil mixtures.

Soil mixture	NH4	NO3	Total min N	Total N	Total C
	(µg g^{-1})			(g kg^{-1})	
grassland (G)	8.634	58.094	66.728	2.689	26.830
agricultural (A)	0.267	102.370	102.637	4.368	47.161

Table S2. Effects of N treatment and depth on N content of the two soil mixtures at harvest calculated with ANOVA analyses. F values are presented. Significant results are indicated in bold ($P < 0.05$), arcsine transformation is indicated as †.

	Grassland soil mixture		Agricultural soil mixture	
	Total N (g kg^{-1})	Mineral N (mg kg^{-1}) †	Total N (g kg^{1})	Mineral N (mg kg^{1})
N	1.9, (0.192)	42.9, (**<0.001**)	0.04, (0.842)	0.4, (0.533)
Depth	4.7, (**0.030**)	3.0, (0.086)	0.5, (0.606)	1.0, (0.411)
N:Depth	0.2, (0.838)	1.0, (0.382)	0.7, (0.515)	0.3, (0.769)

Table S3. Plant growth metrics across soil mixtures and nitrogen (N) treatments. Means and standard deviations (n=3) are shown. H = high N treatment, L = low N treatment. Significant differences between N treatments within each soil mixture are indicated by letters ($P < 0.05$). ns: non-significant.

Soil mixture	N	Shoot dry weight (g)	SPAD	Shoot total N (g kg^{-1})	Total plant N (g)	Chlorophyll (µg mL^{-1})
Grassland	HN	6.80 ± 0.93 a	44.13 ± 0.55 a	37.58 ± 5.65 a	0.26 ± 0.06 ns	28.49 ± 0.89 a

	LN	1.67 ± 0.36 b	26.27 ± 2.90 b	23.60 ± 12.55 b	0.04 ± 0.01 ns	8.20 ± 2.00 b
Agricultural	HN	8.34 ± 1.66 ns	46.17 ± 4.00 ns	35.78± 3.55 ns	0.30 ± 0.08 ns	23.84 ± 3.81 ns
	LN	10.93 ± 2.32 ns	48.17 ± 4.06 ns	37.18 ± 1.70 ns	0.41 ± 0.08 ns	29.25 ± 3.53 ns

Table S4. Effects of nitrogen (N) and depth on architectural phenotypes calculated with ANOVA. Significant results are indicated in bold (*P* < 0.05). Mean values and standard deviations for total root length (TRL), lateral root length (LRL), axial root length (ARL), lateral root branching density (LRBD) and specific root length (SRL). are provided in Fig. S1. LRBD data of the agricultural soil mixture were arcsine-transformed to meet ANOVA assumptions.

		TRL (cm) [1]	LRL (cm) [1]	ARL (cm)	LRBD ($\#*cm^{-1}$)	SRL ($m*g^{-1}$)
Agricultural soil mixture						
N	P	**0.025**	**0.025**	**0.046**	0.149	0.095
	F	6.1	6.1	4.7	2.5	3.1
Depth	P	0.661	0.4	0.254	0.185	**< 0.001**
	F	0.5	1	1.5	2.5	16.9
N:Depth	P	0.072	0.112	**0.009**	0.612	0.219
	F	2.8	2.3	5.4	0.5	1.6
Grassland soil mixture						
N	P	0.492	0.361	0.431	0.051	0.53
	F	0.5	0.9	0.7	4.4	0.4
Depth	P	0.563	0.726	**0.017**	0.189	**< 0.001**
	F	0.7	0.4	4.6	1.8	15.2
N:Depth	P	0.053	**0.043**	**0.026**	**0.042**	0.116
	F	3.2	3.4	4	3.4	2.3

Table S5. Root anatomical phenotypes shown as means ± standard. Bolded values indicate significant differences between the two nitrogen levels within the same soil mixture calculated with T-test. Root Cross-Section Area (RXSA), total cortical area (TCA), living cortical area

(LCA), percentage of LCA of total RXSA (pLCA), aerenchyma area (AA), percentage of AA of total RXSA (RCA), total stele area (TSA).

N	RXSA (mm^2)	TCA (mm^2)	LCA (mm^2)	pLCA (%)	AA (mm^2)	RCA (%)	TSA (mm^2)
Grassland soil mixture							
HN	**1.71 ± 0.14**	**1.41 ± 0.11**	**0.81 ± 0.12**	57.97 ± 7.57	0.09 ± 0.07	6.28 ± 4.46	**0.29 ± 0.04**
LN	**1.00 ± 0.10**	**0.83 ± 0.08**	**0.46 ± 0.06**	55.90 ± 6.14	0.03 ± 0.04	3.61 ± 4.35	**0.17 ± 0.02**
Agricultural soil mixture							
HN	**1.67 ± 0.12**	1.38 ± 0.12	0.71 ± 0.13	51.23 ± 6.62	0.16 ± 0.9	12.06 ± 6.46	0.29 ± 0.04
LN	**1.81 ± 0.10**	1.50 ± 0.09	0.75 ± 0.17	49.84 ± 8.47	0.29 ± 0.15	13 ± 10.52	0.31 ± 0.02

Table S6. Effects of soil mixture, nitrogen (N) treatment, compartment, and sampling location on alpha prokaryotic diversity. Observed richness and Shannon index assessed by PERMANOVA; degrees of freedom for each factor and error term are given in parentheses. Main factors are soil mixture (grassland mixture, agricultural mixture), N treatment (high, low), compartment (bulk soil, rhizosphere, root tissue) and sampling location (for rhizosphere and root tissue samples: seminal roots, lateral roots, roots 0 - 20 cm, roots 20 - 70 cm, 70 - 150 cm; for bulk soil: 0-20 cm, 20-70 cm, 70-150 cm). Values represent the pseudo-F ratio (F), the permutation-based level of significance (*P*) and the R^2. Values at *P*<0.05 are shown in bold.

Factor	Observed richness			Shannon index		
	F	R^2	*P*	F	R^2	*P*
**Soil mixture ($F_{1, 154}$)**	**425.9**	**0.197**	**0.001**	**27.7**	**0.042**	**0.001**
Nitrogen ($F_{1, 154}$)	3.7	0.002	0.052	0.2	0.000	0.664
**Compartment ($F_{2, 154}$)**	**690.2**	**0.638**	**0.001**	**226.8**	**0.681**	**0.001**
Sampling location ($F_{6, 154}$)	0.9	0.002	0.547	1.7	0.015	0.120
Soil mixture x nitrogen ($F_{1, 154}$)	0.6	0.000	0.458	0.1	0.000	0.765
**Soil mixture x compartment ($F_{2, 154}$)**	**107.6**	**0.099**	**0.001**	**21.8**	**0.066**	**0.001**
Nitrogen x compartment ($F_{2, 154}$)	0.5	0.000	0.576	0.0	0.000	0.998
Soil mixture x sampling location $_{(F6, 154)}$	0.5	0.001	0.772	0.4	0.004	0.887
Nitrogen x sampling location $_{(F6, 154)}$	0.4	0.001	0.881	0.2	0.002	0.959
Compartment x sampling location $_{(F4, 154)}$	1.0	0.002	0.400	0.6	0.004	0.647
Soil mixture x nitrogen x compartment ($F_{2, 154}$)	1.5	0.001	0.254	0.5	0.002	0.612
Soil mixture x nitrogen x sampling location $_{(F6, 154)}$	0.9	0.002	0.508	1.4	0.012	0.230
Soil mixture x compartment x sampling location $_{(F4, 154)}$	1.4	0.003	0.243	1.2	0.007	0.338
Nitrogen x compartment x sampling location $_{(F4, 154)}$	0.4	0.001	0.834	0.3	0.002	0.851
Soil mixture x nitrogen x compartment x sampling location $_{(F4, 154)}$	1.1	0.002	0.321	1.5	0.009	0.173

2. Supplementary figures

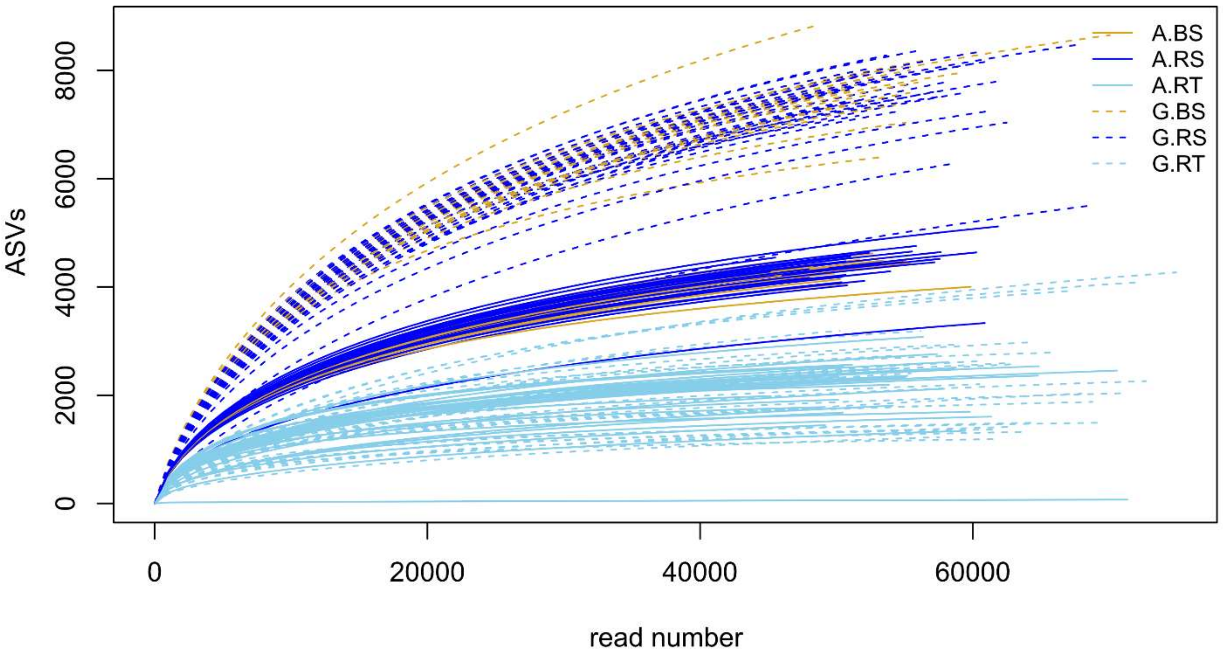

Fig. S1. Rarefaction curves by compartment and soil mixture. A: agricultural soil mixture; G: grassland soil mixture; BS: bulk soil; RS: rhizosphere; RT: Root tissue. ASVs: Amplicon sequence variance.

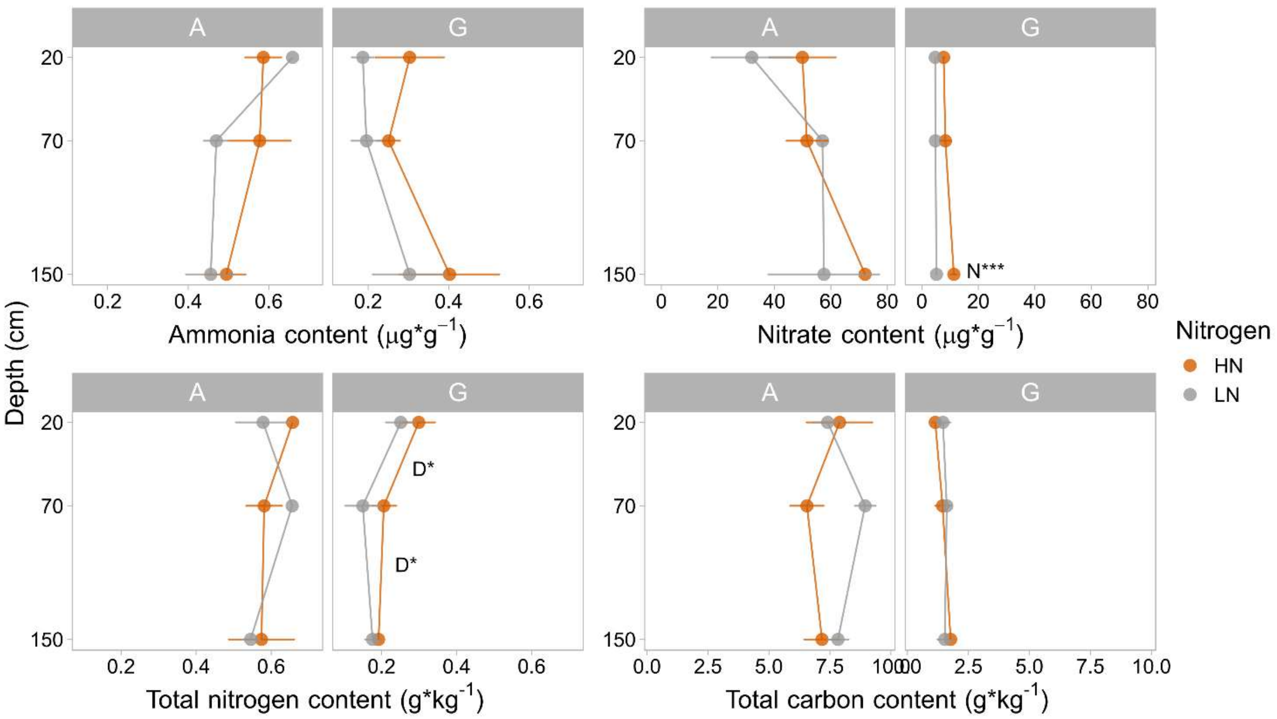

Fig. S2. Nitrogen (N) and carbon content in the two soil mixtures by depths at plant harvest. Mean values and standard errors (n=3) are shown. Posthoc Tukey's Honestly Significant Difference test results between either N levels (HN: high nitrogen, LN: low nitrogen), or between depths (D) are shown in black as *P* values: <0.1 (˙); <0.05 (*); <0.0005 (***). The absence of *P* values indicates no significant difference. A: agricultural soil mixture. G: Grassland soil mixture.

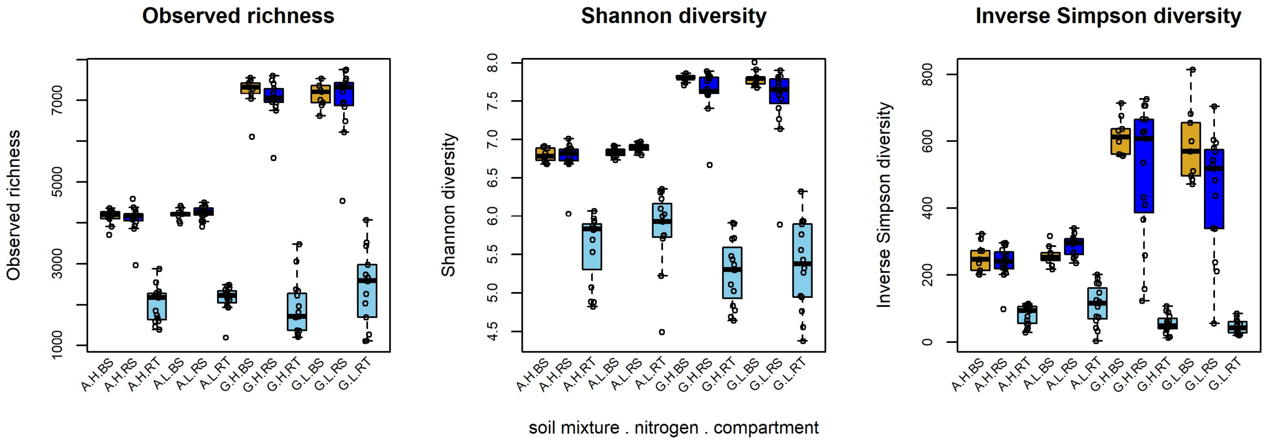

Fig. S3. Alpha diversity per compartment. A: agricultural soil mixture; G: grassland soil mixture; BS: bulk soil; RS: rhizosphere; RT: Root tissue; H: high N; L: low N.

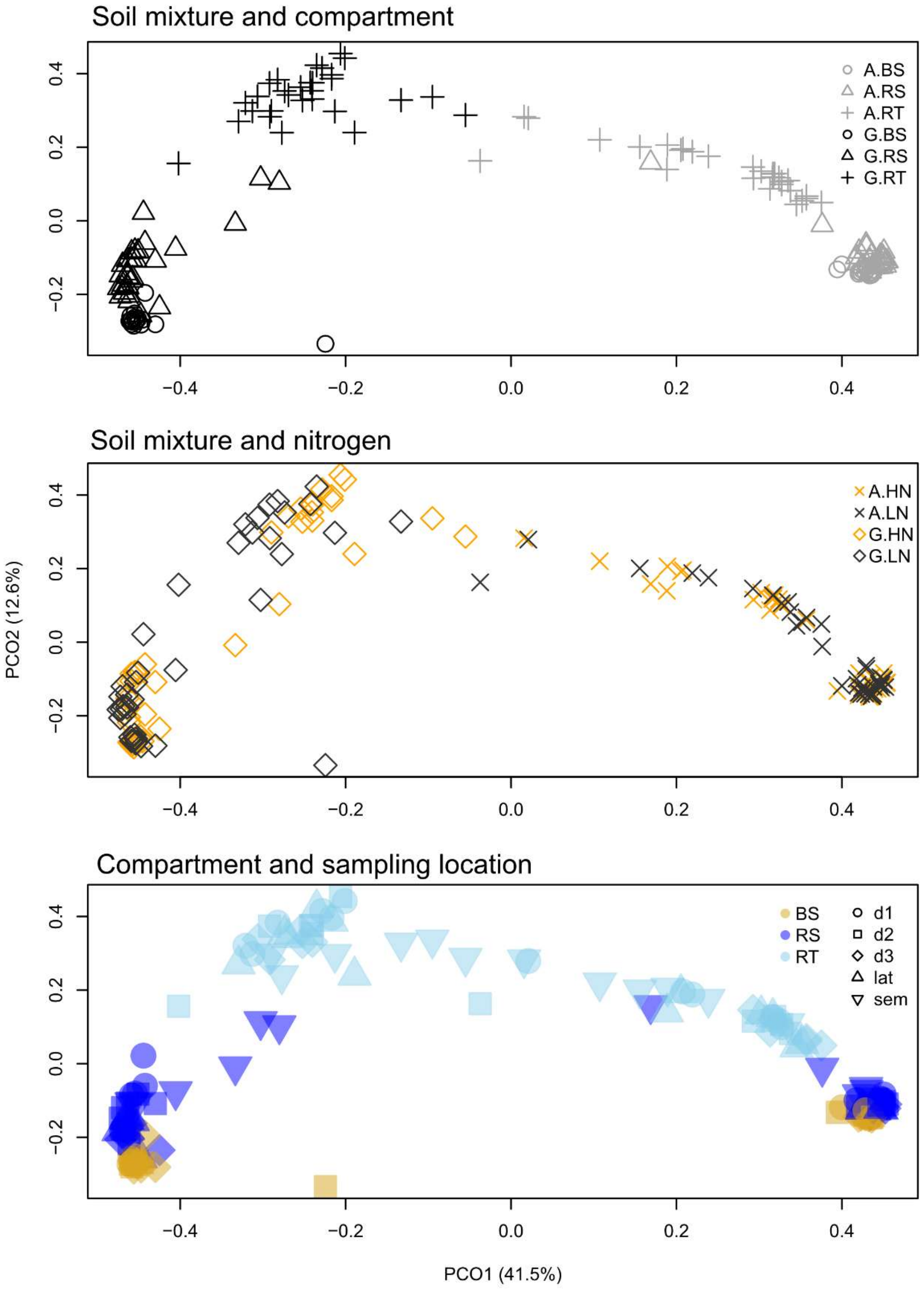

Fig. S4 Separation of root prokaryotic community structure by the different experimental factors using unconstrained (principal coordinate ordinations, PCoA) multivariate analyses on the median values of Bray-Curtis dissimilarities. The three panels show the same ordination, with different factors coded to colors or to shapes. Each point represents a sample. G (grassland soil mixture) or A (agricultural soil mixture); and by compartment: BS (bulk soil), RS (rhizosphere) and RT (root tissue). Nitrogen (N) treatments: H (high N) or L (low N). Sampling location; for rhizosphere and root tissue samples: seminal roots (sem), lateral roots (lat), roots 0 - 20 cm (d1),

roots 20 - 70 cm (d2), 70 - 150 cm (d3); for bulk soil: 0-20 cm (d1), 20-70 cm (d2), 70-150 cm (d3). The variance explained by each PCoA axis is given in brackets.

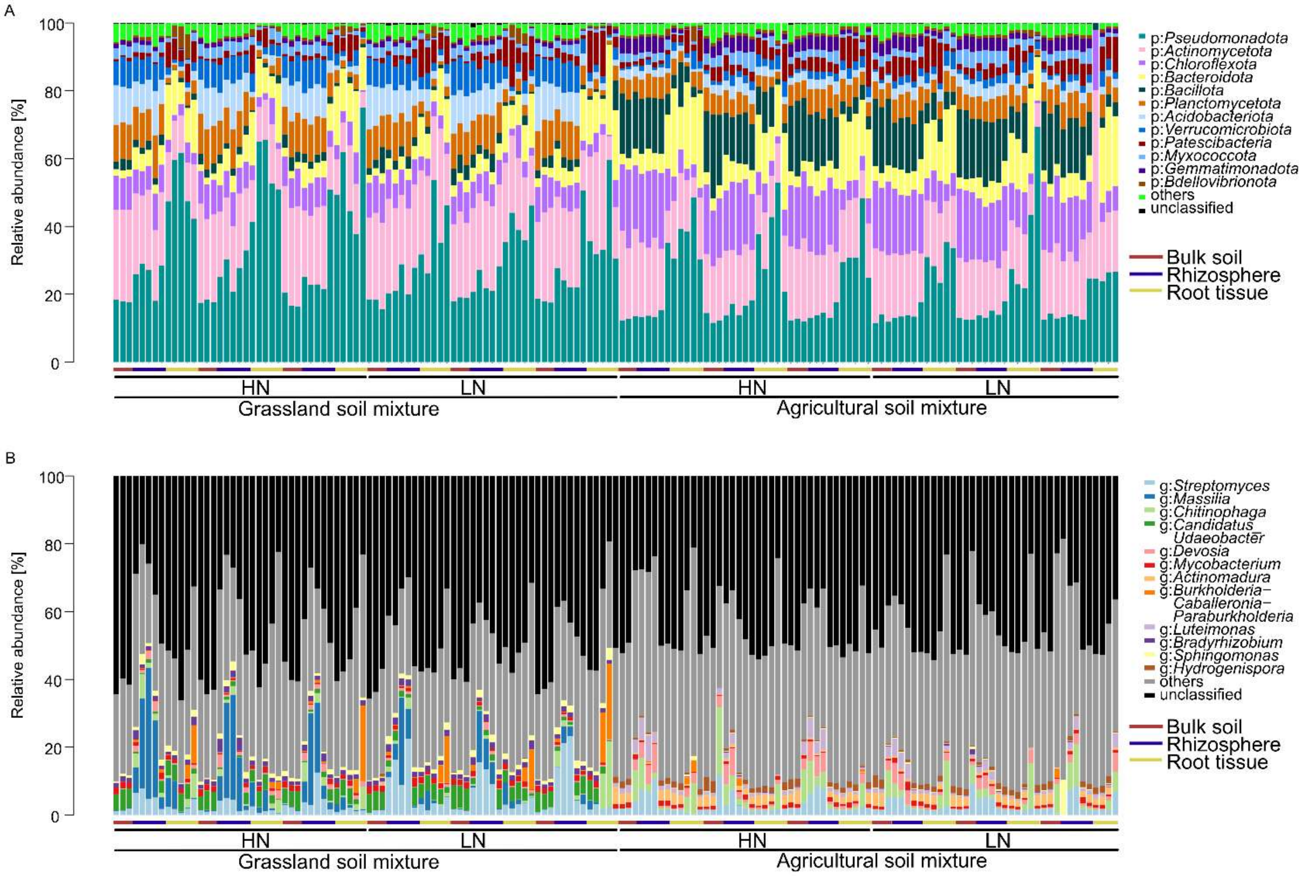

Fig. S5. Relative abundance of major prokaryotic phyla (A) and genera (B) across the 155 different samples. Relative abundances were calculated on rarefied read counts. Individual samples are labeled by soil mixture (grassland or agricultural soil mixture), nitrogen (N, H: high or L:low) and compartment (indicated by colored horizontal lines - bulk soil, rhizosphere, root tissue).

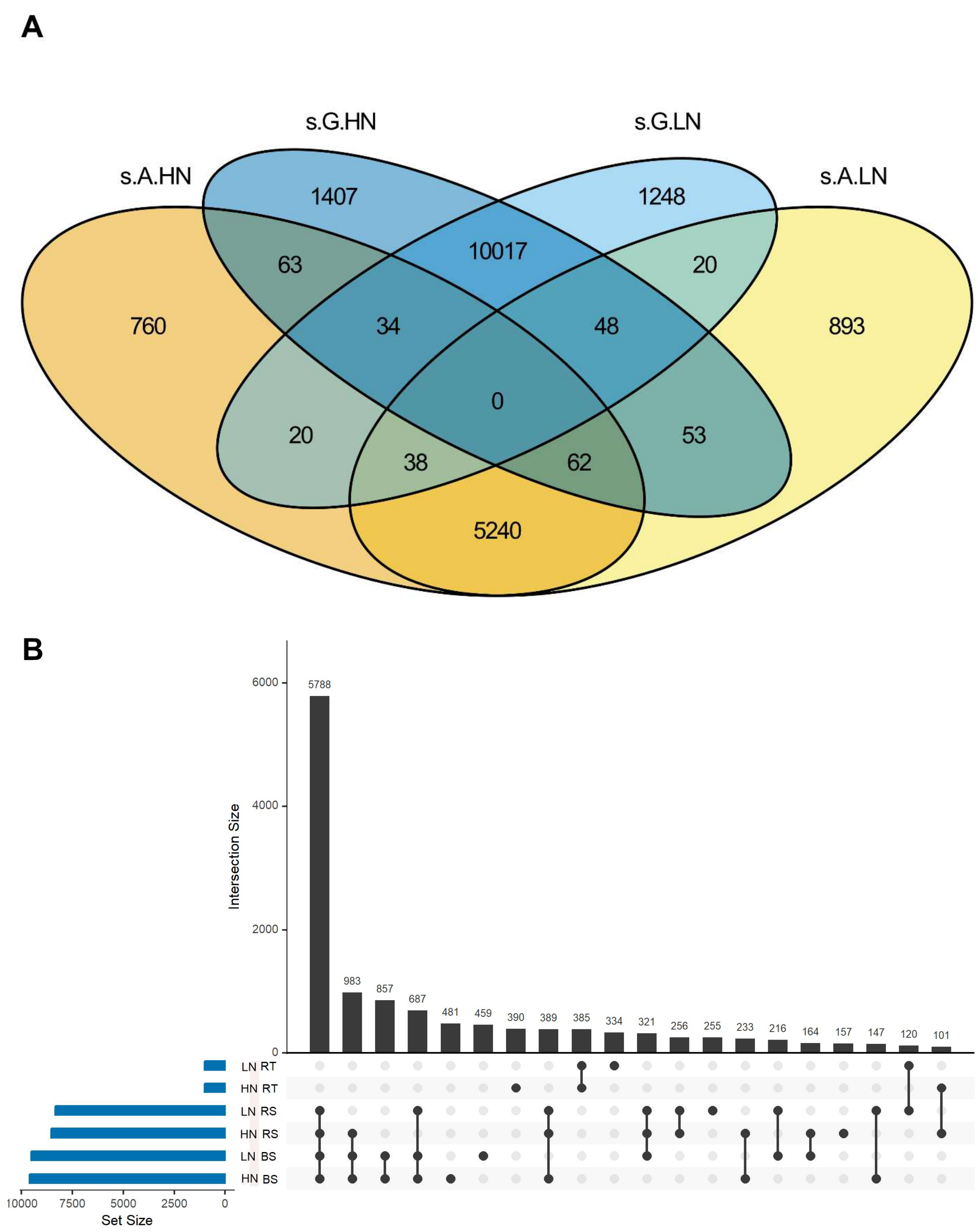

Fig. S6. Indicator species statistics by soil mixture and nitrogen (N) levels, and across the N levels and compartments. **A.** Venn diagram of the significant ASVs in each soil mixture and nitrogen level, and their combinations. B. UpSet plot showing the overlap of significant ASVs (q < 0.05) across nitrogen levels and compartments. Each horizontal bar on the left represents the total number of significant ASVs identified in a specific group, defined by N treatment (high -HN or low -LN) and compartment (bulk Soil, BS; rhizosphere, RS; or root tissue, RT). The

vertical bars at the top indicate the number of ASVs shared between group combinations, as shown by the connected dots below each bar. This visualization highlights both unique and shared microbial indicators across the combination of N and compartments.

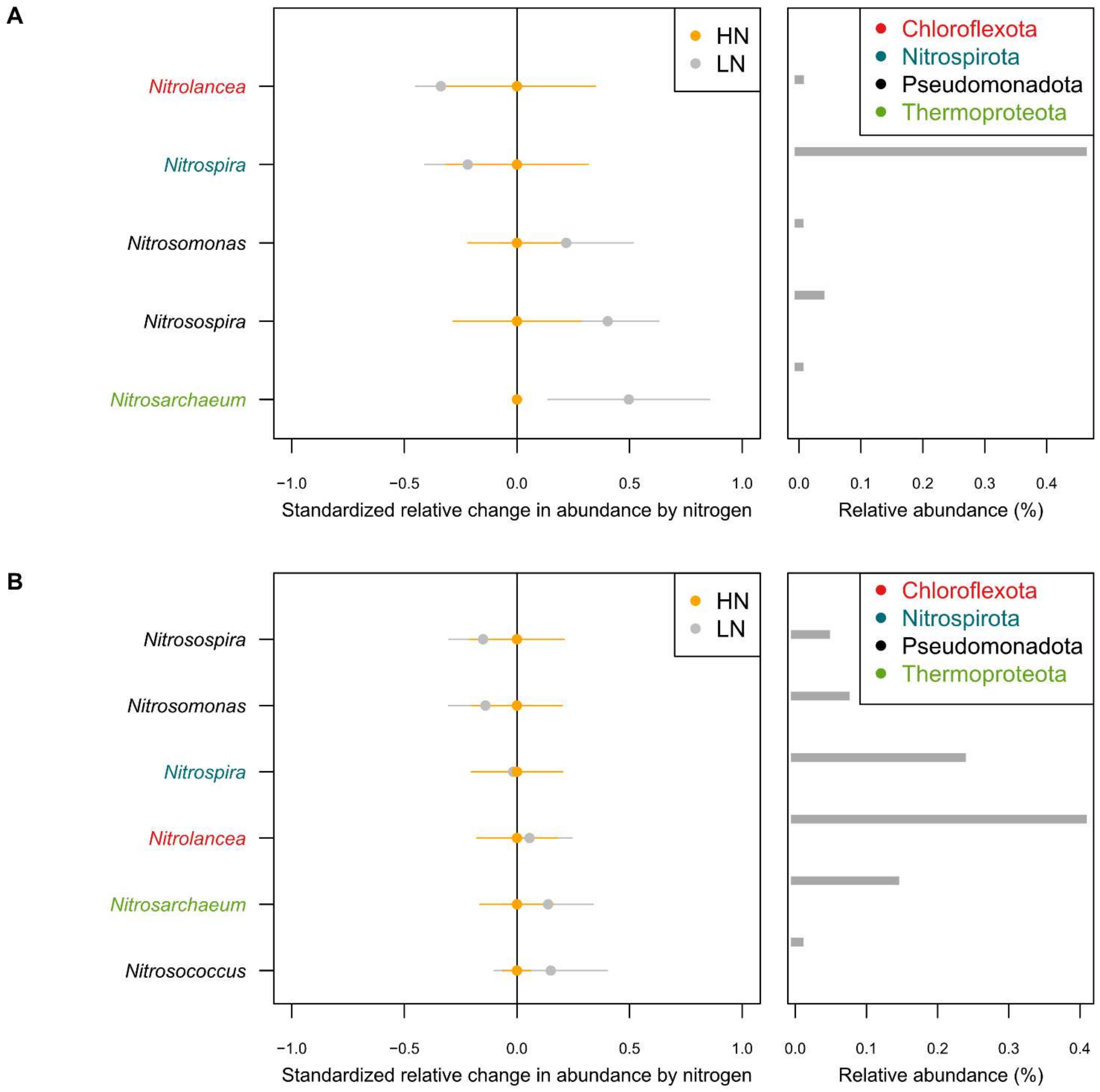

Fig. S7. Ammonia oxidizing genera, plus the nitrite-oxidizing bacteria *Nitrolancea* and their relative change and relative abundance in the rhizosphere of plants growing in the grassland mixture (**A**) and the agricultural soil mixture (**B**). Genus names are color-coded by phyla. The standardized relative mean difference between low N (LN) and high N (HN) and standard errors are provided for each taxon. Relative abundance per genus as percentage of the subset dataset by soil mixture and compartment, is shown on the right.

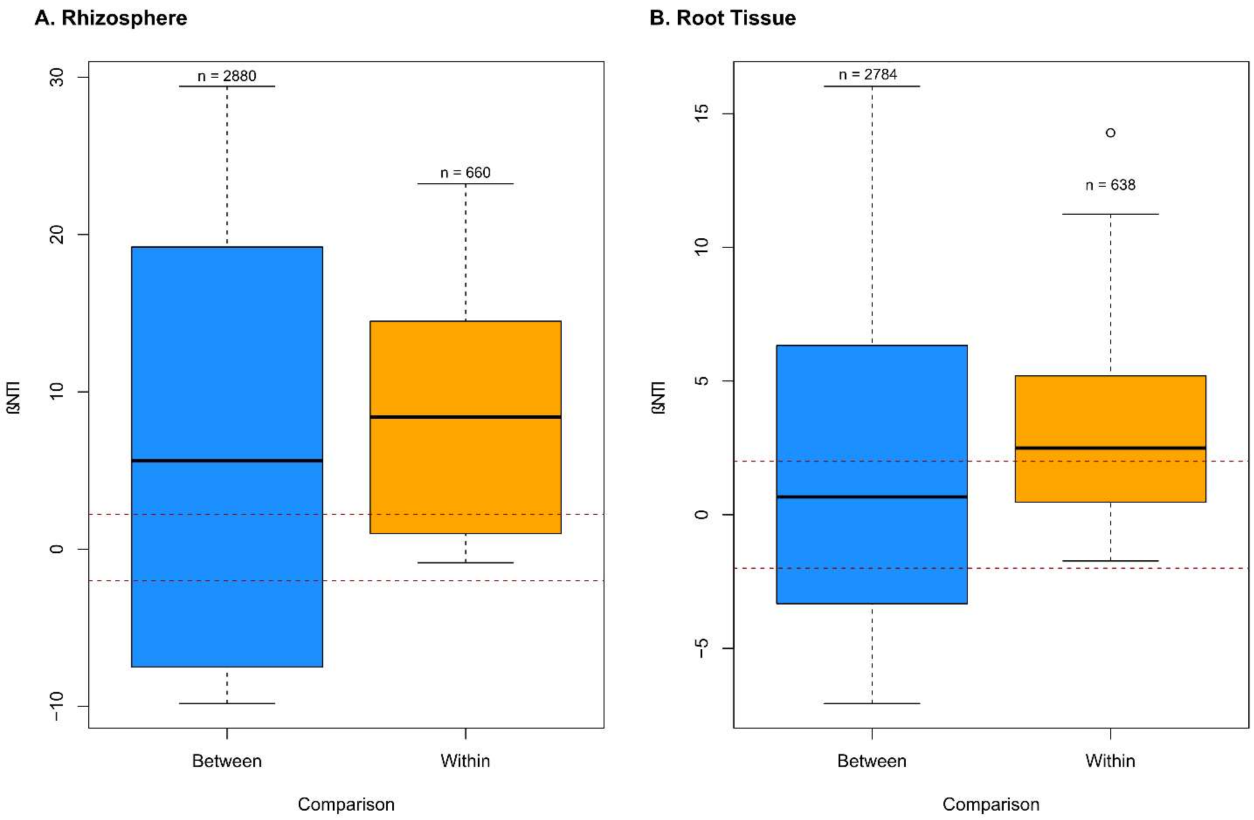

Fig. S8. Dominant community assembly processes and phylogenetic signatures by sampling location measured through the distribution of pairwise Beta Nearest Taxon Index (βNTI) of rhizosphere (A) and root tissue (B), in the two soil mixtures (agricultural and grassland). βNTI quantifies phylogenetic turnover between (in blue) and within (in yellow) communities at the different sampling locations along the root. Values above +2 (upper dashed red line) suggest variable selection or dispersal limitation driving phylogenetic signature of the community to be more distantly related to each other than would be expected by chance; values between -2 and +2 suggest a phylogenetic signature dominated by stochastic processes. The horizontal line within each box represents the median, the box boundaries indicate the interquartile range (IQR, 25th to 75th percentile), and the whiskers extend to 1.5 times the IQR from the box edges. Circles represent outliers. The number of comparisons is indicated for each boxplot.

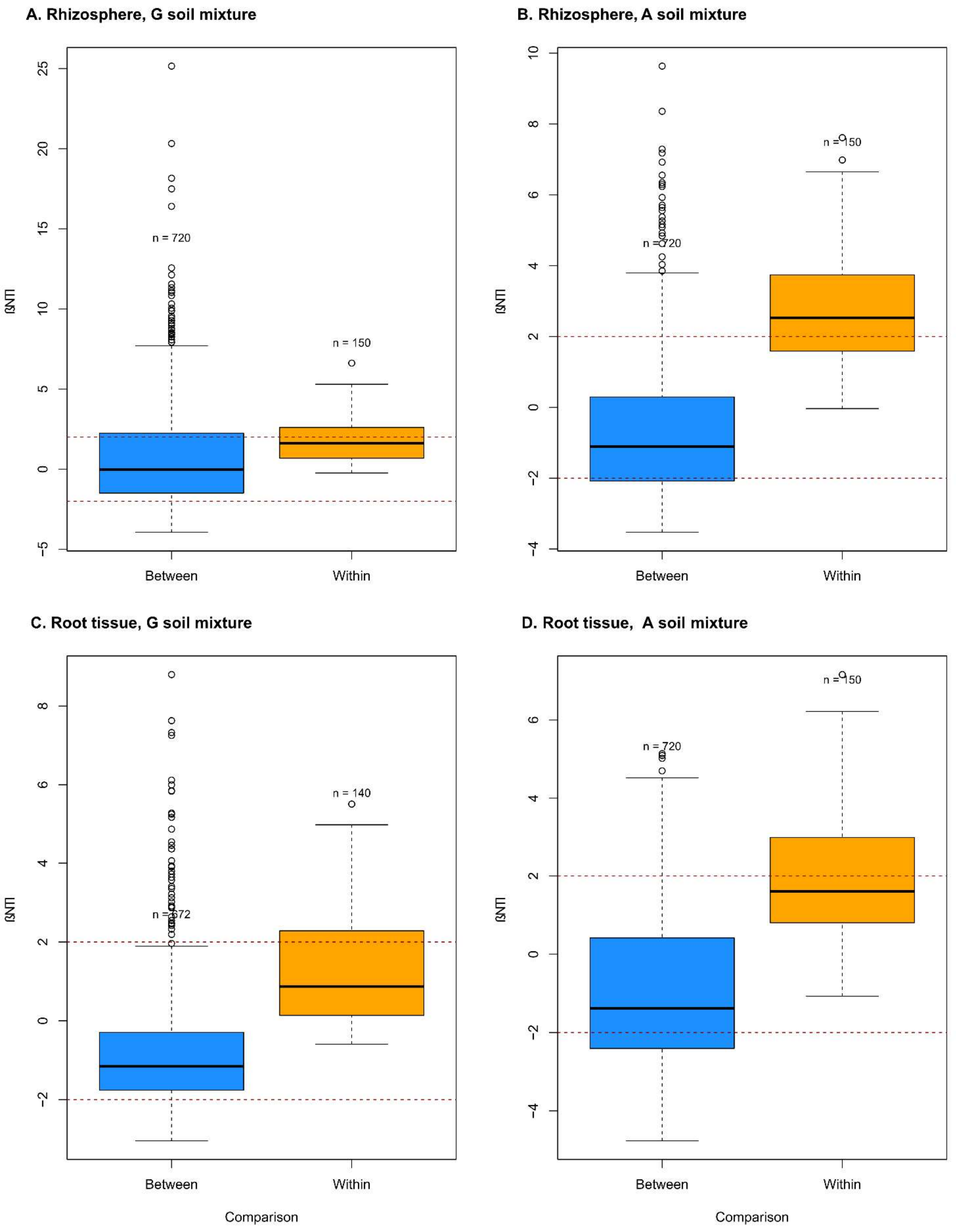

Fig. S9. Dominant community assembly processes and phylogenetic signatures by sampling location measured through the distribution of pairwise Beta Nearest Taxon Index (βNTI) split by rhizosphere (panels A and B) and root tissue (panels C and D) for each soil mixtures (grassland, G mixture (panels A, C) and agricultural (panels B, D)). βNTI quantifies phylogenetic turnover

between (in blue) and within (in yellow) communities at the different sampling locations along the root. Values above +2 (upper dashed red line) suggest variable selection or dispersal limitation driving phylogenetic signature of the community to be more distantly related to each other than would be expected by chance; values between -2 and +2 suggest a phylogenetic signature dominated by stochastic processes. The horizontal line within each box represents the median, the box boundaries indicate the interquartile range (IQR, 25th to 75th percentile), and the whiskers extend to 1.5 times the IQR from the box edges. Circles represent outliers. The number of comparisons is indicated for each boxplot.

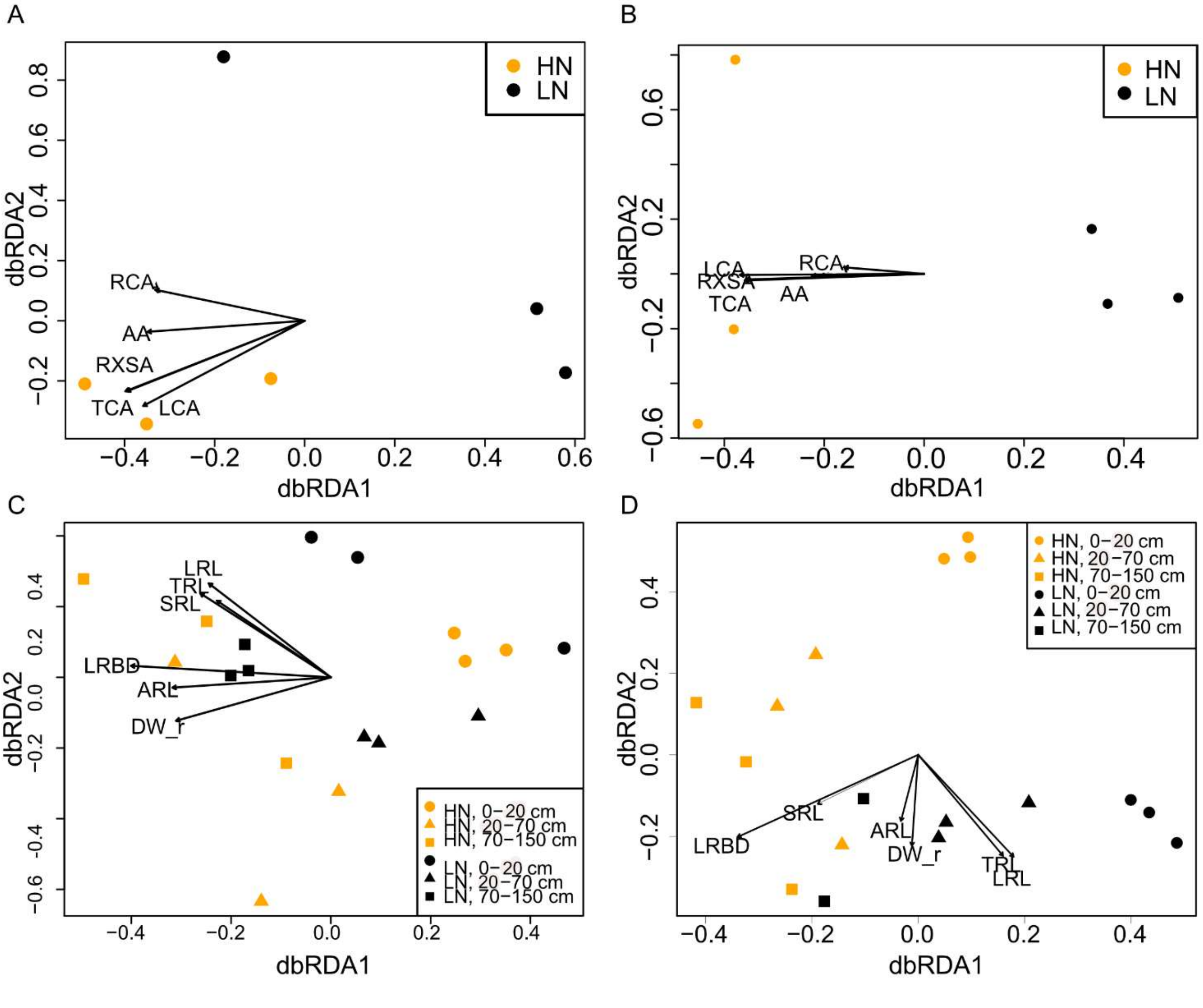

Fig. S10. Distance-based redundancy analyses of anatomical (A, B) and architectural (C, D) root phenotypes as predictors of beta diversity based on the median values of Bray-Curtis dissimilarities of A, C rhizosphere and B, D root tissue. Architectural phenotypes were measured at three depths and include: LRL: Lateral root length; TRL: total root length; ALR: Axial root length; LRBD: lateral root branching density; DW_r: dry weight; SRL: Specific root length. Anatomy was measured on one root segment per plant, collected above 20 cm, and include: AA:

Cortical aerenchyma area, RCA: percentage of cortical area that is aerenchyma; TCA: total cortical area, RXSA: Root cross-section area; LCA: living cortical area. Significant phenotypes according to PERMANOVA are shown with their R^2 and *P* values in Table 3.

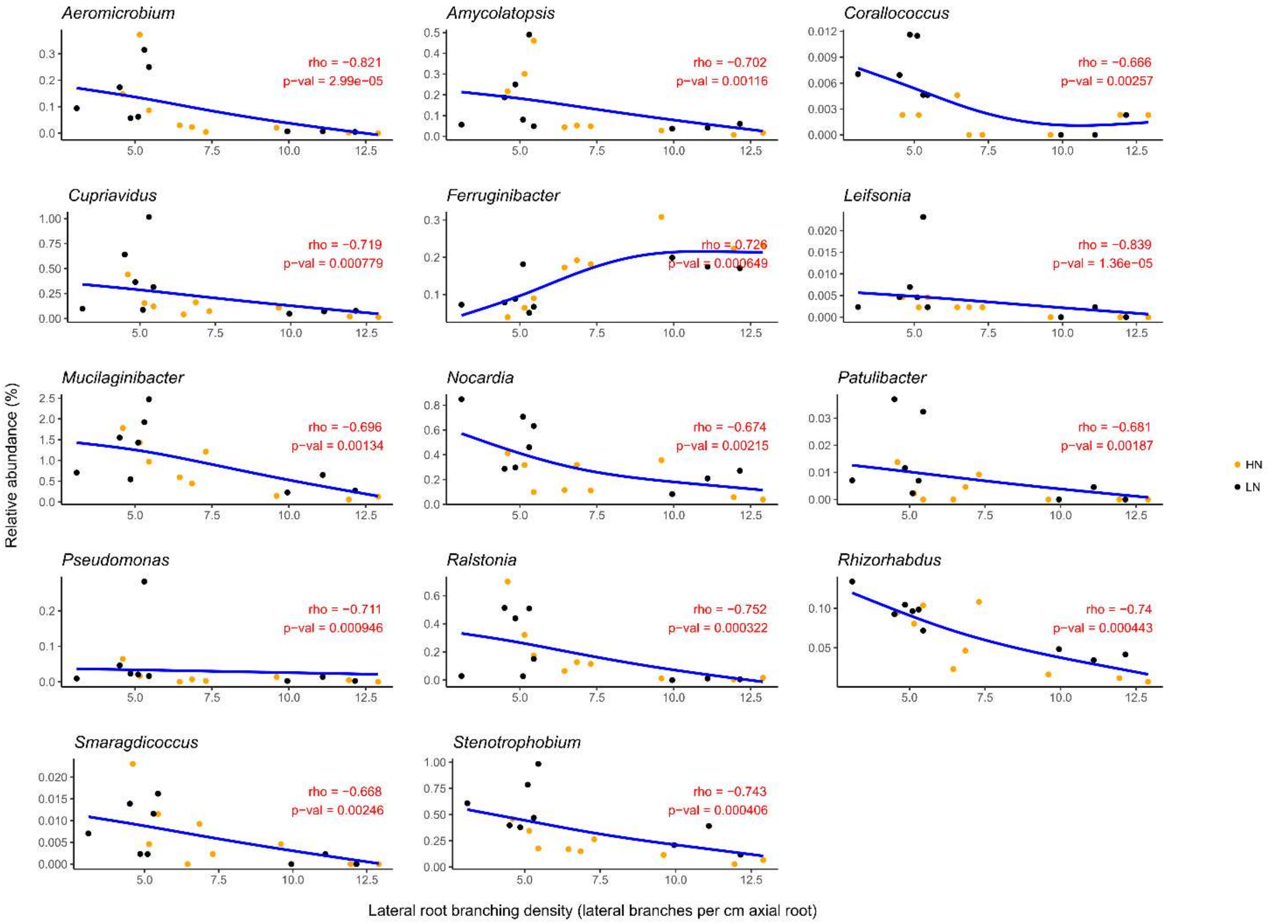

Fig. S11. Scatterplots of lateral root branching density vs. relative abundance of genera in the rhizosphere of the grassland soil mixture. The relative abundance was calculated as percentage of the total abundance of all the genera found in the grassland soil mixture in the rhizosphere, on genus-aggregated and filtered ASVs (removing rare ASVs with a relative abundance ≤ 0.01%, sparse ASVs not occurring in at least three samples, and outlier ASVs that exceed the abundance in the second most abundant sample by 100-fold). Only genera having Spearman correlations with $q < 0.05$ are presented. Lines show generalized additive model adjustments between the two variables. Rho and *P*-values of the Spearman correlations are provided. Dots color-coded by nitrogen treatment.

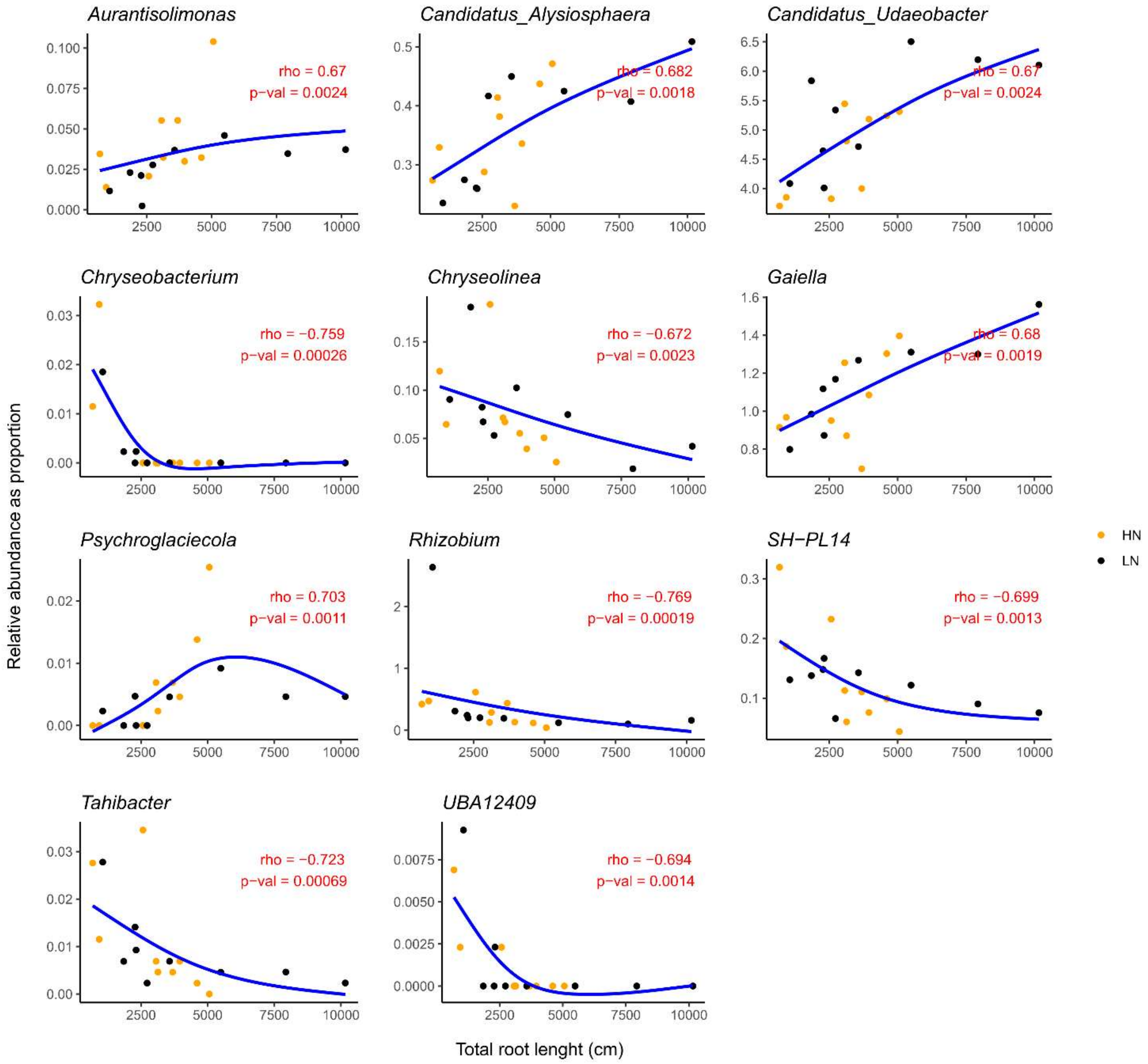

Fig. S12. Scatterplots of total root length vs. relative abundance of genera of the rhizosphere in grassland soil mixture. The relative abundance was calculated as percentage of the total abundance of all the genera found in the grassland soil mixture in the rhizosphere, on genus-aggregated and filtered ASV (removing rare ASVs with a relative abundance ≤ 0.01%, sparse ASVs not occurring in at least three samples, and outlier ASVs that exceed the abundance in the second most abundant sample by 100-fold). Only genera having Spearman correlations with $q < 0.055$ are presented. Lines show generalized additive model adjustments between the two variables. Rho and P-values of the Spearman correlations are provided. Dots color-coded by nitrogen treatment.

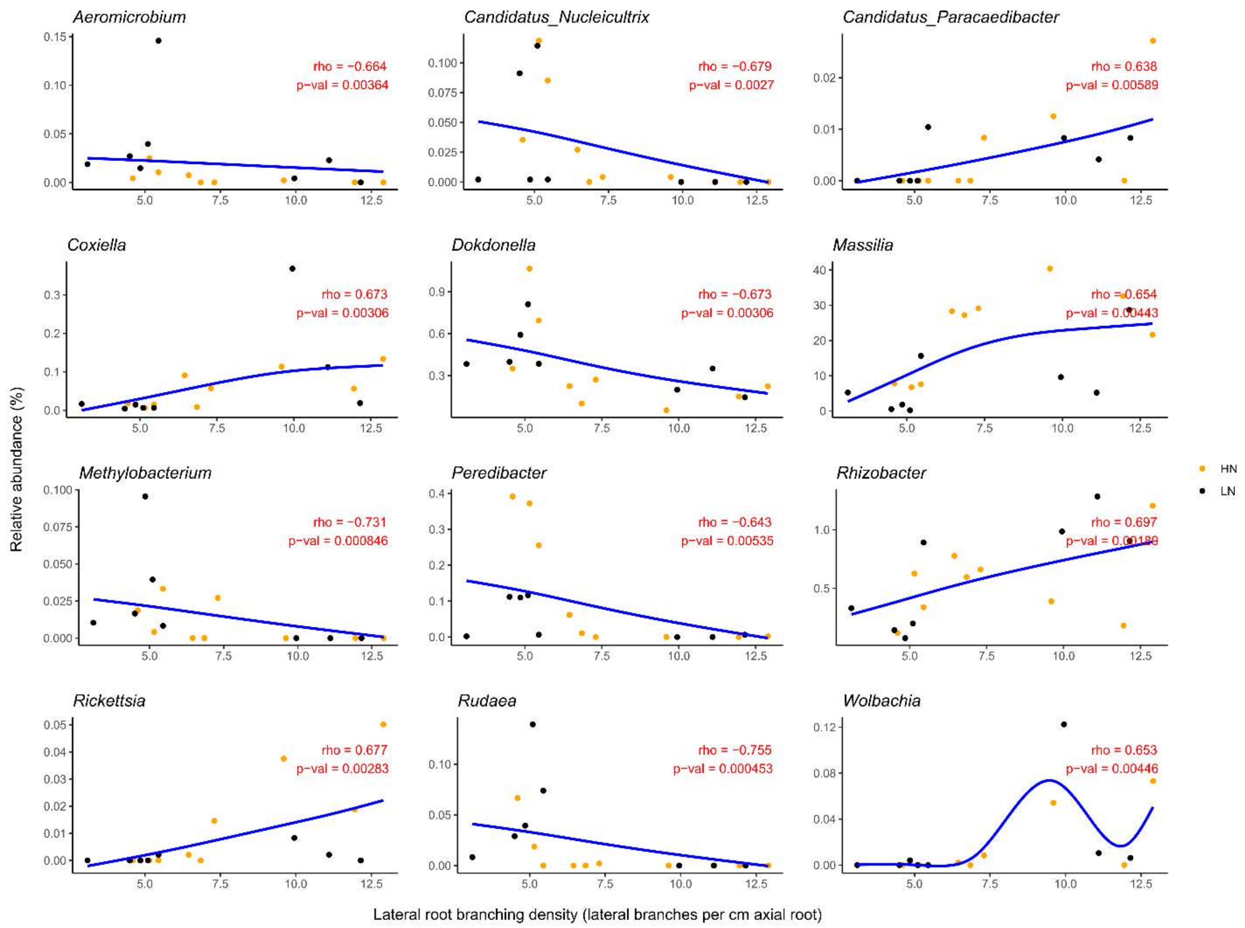

Fig. S13. Scatterplots of lateral root branching density vs. relative abundance of genera in the root tissue of the grassland soil mixture. The relative abundance was calculated as percentage of the total abundance of all the genera found in the grassland soil mixture in the root tissue, on genus-aggregated and filtered ASV (removing rare ASVs with a relative abundance ≤ 0.01%, sparse ASVs not occurring in at least three samples, and outlier ASVs that exceed the abundance in the second most abundant sample by 100-fold). Only genera having Spearman correlations with $q < 0.09$ are presented in the graph. Lines show generalized additive model adjustments between the two variables. Rho and *P*-values of the Spearman correlations are provided. Dots color-coded by nitrogen treatment.

3. Supplementary information

Statistical analyses followed to measure ecological processes shaping microbial community assembly by sampling location in the rhizosphere and endosphere

We calculated the beta-nearest taxon index (βNTI) using the *iCAMP* package (v1.5.12) (Ning *et al.*, 2020), following the approach described by Stegen *et al.* (2012), and used recently by Larsen *et al.* (2023).

First, a phylogenetic tree was constructed from the filtered (see filters applied in the Methods section of the main text of this paper) ASV sequences. Sequences were aligned using the *AlignSeqs* function from the DECIPHER v11.33 package (Wright, 2016). The aligned sequences were then converted to a phyDat object. Phylogenetic distances were calculated using maximum likelihood (function *dist.ml* from *phangorn* v2.12.1.3 (Schliep, 2011)), and an initial phylogenetic tree was inferred using the Neighbor-Joining (NJ) method with the function *NJ* from *ape* v5.8-1 (Paradis and Schliep, 2018). This tree was then optimized under a General Time Reversible (GTR) model, accounting for invariant sites and gamma-distributed rates with the function *optim.pml* from *phangorn*, to obtain the final tree.

Subsequently, βNTI was calculated to assess the phylogenetic turnover between samples. Pairwise phylogenetic distances between ASVs were computed using the function *pdist.big*, from *iCAMP*, which utilizes a large memory approach for efficiency, saving temporary files to the working directory. The βNTI values were then calculated using the function *bNTI.big*, from *iCAMP*. This calculation considered the community abundance values, a metadata grouping variable (sampling location), and the calculated phylogenetic distances. The analysis was performed with 4 parallel workers, allocated 20 GB of memory, was weighted by ASV abundances, and included 999 randomizations for null model comparison.

The resulting βNTI matrix was visualized as a heatmap to display pairwise phylogenetic turnover. To statistically assess differences in assembly processes, βNTI values were categorized into "Within" and "Between" group comparisons based on sampling location. Data manipulation for this categorization utilized functions from the dplyr (v1.1.4) package. A Wilcoxon rank-sum test was performed to compare the distribution of "Within" versus "Between" βNTI values, testing the alternative hypothesis that "Within" group βNTI values are less than "Between" group βNTI values. Finally, the distributions of "Within" and "Between" βNTI values were visualized using boxplots.

References

Paradis E, Schliep K. 2018. ape 5.0: an environment for modern phylogenetics and evolutionary analyses in R. *Bioinformatics,* **35**: 526-528.

Schliep KP. 2011. phangorn: phylogenetic analysis in R. *Bioinformatics,* **27**: 592-593.

Wright ES. 2016. Using DECIPHER v2. 0 to analyze big biological sequence data in R.

Supplementary video. Experimental setup and sampling provided as MP4.
